# Groundbreaking Insights Into SIRT1/NRF2‐Mediated Ferroptosis Inhibition by Resveratrol in Parkinson's Disease Models

**DOI:** 10.1111/cns.70648

**Published:** 2025-11-11

**Authors:** Qian Zheng, Dan Huang, Liping Zhao, Xincheng Long, Qiuxia Tu, Lingli Song, Jiaojiao Wang, Wen Zheng, Xiaojun Wen, Chunlin Zhang, Li Lei

**Affiliations:** ^1^ Engineering Research Center for Molecular Medicine, Key Laboratory of Medical Molecular Biology of Guizhou Province, School of Basic Medical Science Guizhou Medical University Guiyang People's Republic of China; ^2^ Department of Neurology Affiliated Hospital of Guizhou Medical University Guiyang People's Republic of China

**Keywords:** ferroptosis, NRF2, parkinson's disease, resveratrol, SIRT1

## Abstract

**Background:**

Parkinson's disease (PD) is the second most prevalent neurodegenerative disorder, characterized by the degeneration of dopamine (DA) neurons in the substantia nigra (SN) of the midbrain. Recent studies have highlighted the role of ferroptosis in neuronal death, with elevated peroxide levels being a hallmark of this process. Resveratrol (RSV), a natural compound, has shown promise as a neuroprotective agent. This study explores the potential of RSV in mitigating ferroptosis in PD and elucidates its mechanisms.

**Methods:**

Network pharmacology was employed to predict the interactions between RSV, ferroptosis, and PD‐related targets. Cytoscape protein‐protein interaction (PPI) analysis identified key potential targets, while Gene Ontology (GO) and Kyoto Encyclopedia of Genes and Genomes (KEGG) pathway enrichment analyses provided insights into the probable mechanisms linking RSV, ferroptosis, and PD. Subsequently, in vitro experiments were conducted to validate these findings, followed by in vivo studies to confirm the therapeutic efficacy of RSV in PD.

**Results:**

Network pharmacology results indicated that RSV, PD and ferroptosis interact at multiple biological levels. Compared to the PD group, RSV upregulated the expression of SIRT1 and NRF2 and alleviated MPTP‐induced motor deficits in mice. Furthermore, RSV reduced levels of MDA, ROS, lipid peroxidation, and cellular iron, while upregulating ferroptosis‐negative regulators such as GPX4 and FTH1, as well as pathway indicators like SIRT1 and NRF2. Inhibition of SIRT1 and NRF2 resulted in a decrease in the expression of GPX4/FTH1 and the SIRT1/NRF2 signaling pathway.

**Conclusions:**

Our findings demonstrate that RSV alleviates motor dysfunction in PD by inhibiting ferroptosis through SIRT1/NRF2 activation, providing novel mechanistic insights into its therapeutic potential for neurodegenerative diseases.

## Graphical abstract

Resveratrol inhibits ferroptosis via the SIRT1/NRF2 pathway in models of Parkinson's disease, including cells, mice, and nematodes. This mechanism highlights its role as a potential neuroprotective agent. Targeting ferroptosis offers a promising therapeutic strategy for Parkinson's treatment.

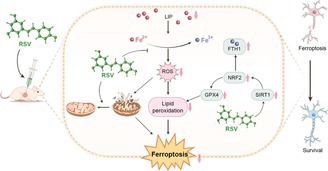

## 1 Introduction

Parkinson's disease (PD), one of the most common neurodegenerative disorders globally, was first described in 1817 and currently affects approximately 2% of individuals over 60 years of age [1]. This progressive neurological condition is pathologically defined by the selective degeneration of dopaminergic (DA) neurons in the substantia nigra pars compacta (SNpc), leading to characteristic motor impairments including resting tremor, bradykinesia, rigidity, and postural instability, along with diverse non‐motor manifestations such as cognitive impairment, autonomic dysfunction, and sleep disorders. The precise mechanisms underlying PD remain complex and multifactorial, with various molecular pathways implicated in its pathology, such as the accumulation of misfolded proteins, mitochondrial dysfunction, oxidative stress, inflammation, and elevated iron levels [2]. Current therapeutic strategies primarily rely on dopaminergic replacement therapy, which effectively manages symptoms but fails to halt disease progression. This critical therapeutic gap underscores the urgent need to identify novel molecular targets for disease‐modifying interventions that could potentially slow or prevent neurodegeneration in PD.

Mounting evidence implicates ferroptosis, a novel iron‐dependent regulated cell death mechanism first characterized by Dixon et al. in 2012 [3], as a critical driver of Parkinson's disease progression. Ferroptosis is regulated by factors that control iron and lipid peroxide homeostasis, and is characterized by the accumulation of lipid peroxides. The Fenton‐like reaction between iron and lipid peroxides produces lipid alkoxyl and hydroxyl radicals, which can generate reactive oxygen species (ROS). These ROS can either initiate further lipid peroxidation (LPO) through free radical chain reactions or directly damage biomolecules, thereby contributing to oxidative stress. Brain tissue, which is particularly vulnerable to oxidative stress, contains high levels of iron and unsaturated fatty acids and consumes approximately 20% of the body's total oxygen supply [4]. Additionally, oligomeric *α*‐synuclein (*α*‐syn) induces the generation of ROS and lipid peroxides within cellular membranes, potentially triggering ferroptosis. One study demonstrated that ferrostatin‐1 (Fer‐1), a specific inhibitor of ferroptosis, protected dopaminergic neurons and improved behavioral deficits in PD models [5]. Therefore, small‐molecule inhibitors of ferroptosis, including iron chelators, ROS scavengers, and activators of the nuclear factor (erythroid‐derived 2)‐like 2 (NRF2) endogenous antioxidant pathway, hold significant therapeutic potential for PD treatment [6].

Natural compounds, known for their structural diversity and broad pharmacological activities, have long served as essential sources for new drug discovery. One such compound is Resveratrol (RSV), a naturally occurring polyphenolic, non‐flavonoid compound found in plants such as grapes, peanuts, blueberries, bilberries, cranberries, and various medicinal herbs. Due to its wide‐ranging health benefits, RSV has garnered significant attention over the past few decades [7]. Extensive studies have confirmed that this phytoalexin exhibits various biological activities, including anti‐inflammatory, antioxidant, and anti‐tumor effects. In PD research, RSV has been shown to alleviate PD symptoms in mice, although the underlying mechanisms remain unclear [8]. Furthermore, RSV has been implicated in modulating cellular ferroptosis in the pathogenesis of several diseases [9, 10], but its role in regulating ferroptosis in PD remains unexplored.

With the rapid advancement of bioinformatics, network pharmacology, leveraging large databases, has become a powerful tool for detailing the mechanisms of complex drug systems, from molecular interactions to pathway analysis. This approach is particularly well suited for studying multi‐target agents, as it integrates various technologies, including systems biology, poly‐pharmacology, molecular network data, bioinformatics, and computer simulation. Numerous studies have utilized network pharmacology to elucidate the mechanisms by which drugs affect disease processes. This method has also emerged as a promising strategy to accelerate drug research and development. However, no comprehensive network pharmacology studies have yet investigated the effects of RSV on ferroptosis in PD.

**FIGURE 1:**
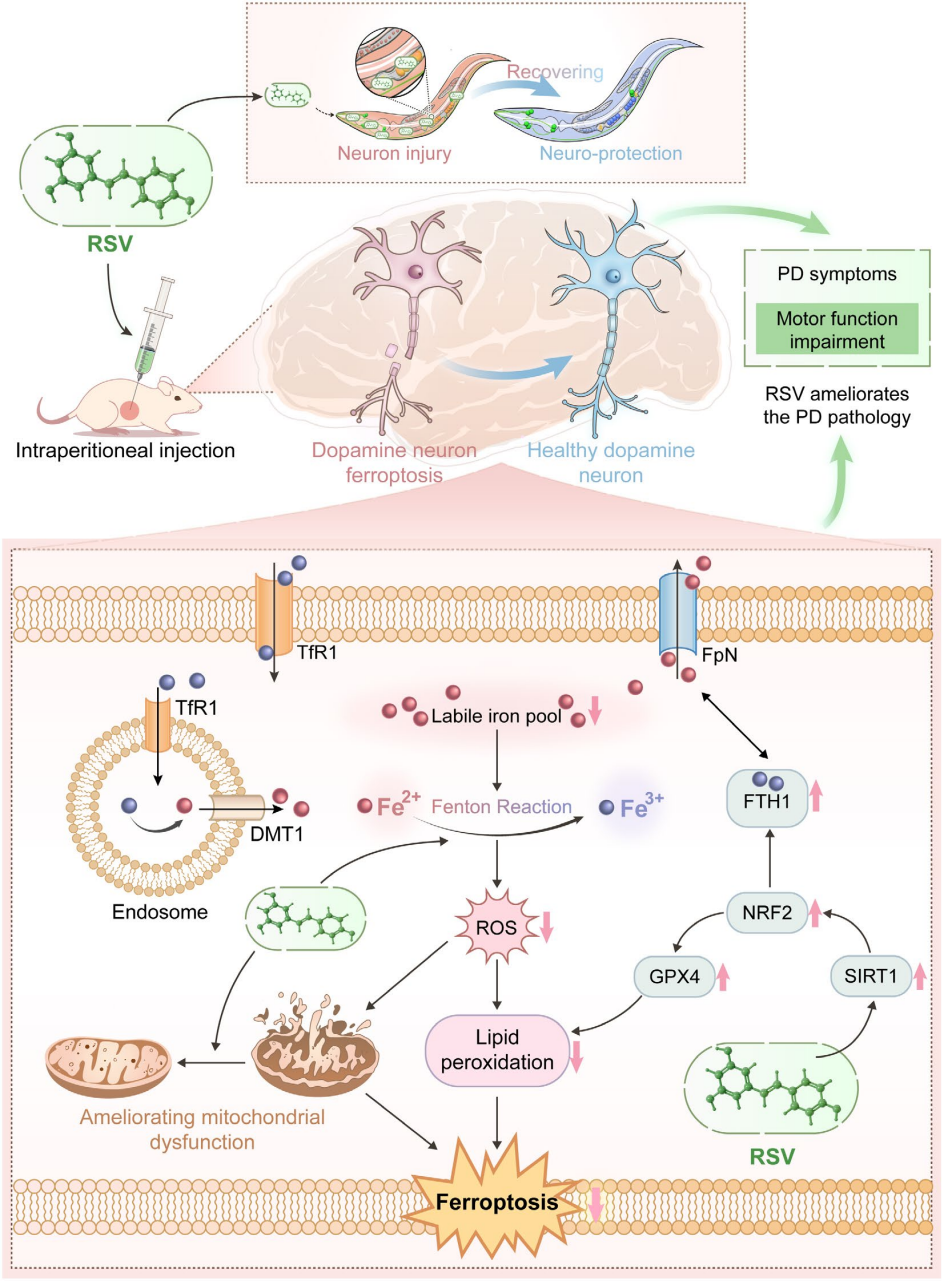
Schematic illustration of the proposed mechanisms underlying RSV‐related anti‐ferroptosis in PD. RSV treatment effectively modulates ROS and iron ion levels, reduces LPO, upregulates GPX4 and FTH1 expression, improves mitochondrial function, and inhibits ferroptosis in PD. The underlying mechanism is likely linked to the SIRT1/NRF2 signaling pathway.

The current study focuses on evaluating the effects of RSV in PD models both in vivo and in vitro (Figure 1). Specifically, the objective is to: (i) validate RSV's anti‐PD effects in vivo/in vitro; (ii) elucidate RSV's anti‐ferroptotic mechanism; (iii) identify core targets via using a combination of network pharmacology and molecular experimental validation.

## 2 Methods

### 2.1 Network Pharmacology

In the network pharmacology analysis, potential therapeutic targets for RSV in the context of PD and ferroptosis were identified through a multi‐database approach. RSV treatment targets were obtained from the HERB database (http://herb.ac.cn/), PD‐related targets from the GeneCards database with a correlation score > 1 (http://www.genecards.org/), and ferroptosis‐related targets from the FerrDb database (http://www.zhounan.org/ferrdb/current/). Overlapping targets were subsequently determined using a Venn diagram to pinpoint potential targets of RSV for PD treatment. To explore protein interactions, both direct and indirect, the STRING database (https://string‐db.org/) was used to analyze these overlapping targets, with species limited to *Homo sapiens* and default settings applied. Cytoscape 3.9.0 was employed to create a protein–protein interaction (PPI) network diagram, and the top 10 core targets were selected based on degree centrality values using Network Analyzer. Further functional and pathway enrichment analyses were performed using R software, focusing on Gene Ontology (GO) and Kyoto Encyclopedia of Genes and Genomes (KEGG) analyses. GO analysis covered three categories: biological process (BP), cellular component (CC), and molecular function (MF), providing insight into the functional roles of the target proteins involved in the pharmacological intervention of PD.

### 2.2 Cell Viability Assay

The viability of PC12 cells was assessed using the MTT assay. Briefly, PC12 cells (1.5 × 10^4^) were seeded into 96‐well plates and pretreated with varying concentrations of RSV (1–50 μM) for 1 h prior to 6‐OHDA exposure (24 h) to assess neuroprotective effects. MTT (10 μM) was then added to each well, and the cells were incubated for 2 h. Absorbance was measured at 490 nm using a microplate reader (BioTek, VT, USA).

### 2.3 Detection of ROS and LiperFluo

Intracellular reactive oxygen species (ROS) and lipid peroxidation (LPO) levels were determined using DCFH‐DA (5 μM) and LiperFluo (10 μM) probes, respectively. PC12 cells in 6‐well plates were treated for 24 h, stained with probes (37°C, 1 h), and visualized using an inverted fluorescence microscope.

### 2.4 Assessment of MDA and GSH Levels

Levels of MDA and GSH were measured using specific assay kits. After treatment, brain tissues from mice were homogenized and sonicated in lysis buffer, followed by centrifugation at 12,000 rpm at 4°C for 30 min. The supernatants were analyzed for MDA and GSH activities using a multimode plate reader.

### 2.5 Mitochondrial Functions

Mitochondrial membrane potential (MMP) was evaluated using the JC‐1 fluorescent probe. PC12 cells (3.5 × 10^4^) were seeded in 6‐well plates overnight, treated for 24 h, and then incubated with JC‐1 according to the manufacturer's protocol. Images were captured using an inverted fluorescence microscope. Healthy mitochondria with high membrane potential emitted red fluorescence, while green fluorescence indicated a decrease in membrane potential. The change in MMP was detected by measuring the fluorescence shift from red to green.

Mitochondrial ROS levels were assessed using MitoSOX Red. Briefly, PC12 cells were seeded in 6‐well plates, treated, and incubated with 10 μM MitoSOX Red at 37°C for 30 min. After washing with PBS, fluorescence was analyzed using laser scanning confocal microscopy.

The morphological structure of mitochondria, including mitochondrial size, shape, and cristae structure, was directly observed by using a JEM‐1400‐FLASH transmission electron microscope (TEM). Briefly, PC12 cells were collected and fixed with 2% glutaraldehyde for 2 h, followed by 1% osmium tetroxide fixation. The cells were dehydrated in a graded ethanol series, embedded in resin and imaged by TEM.

### 2.6 Lifespan Analysis of Nematodes

For the lifespan analysis assay, synchronized L1 larvae (N2) were allowed to grow on NGM plates seeded with *E. coli* OP50 for 1 day at 22°C, reaching the L2 stage to assess lifespan. The nematodes were collected by centrifugation at 3000 rpm for 1 min and then divided into 6‐OHDA (50 mM) lesion groups and control groups (20 mM ascorbic acid) for 1 h. Nematodes in the control and PD groups were transferred to plates seeded with inactive OP50, while those in the RSV group were transferred to plates seeded with 20 mM RSV and inactive OP50 after 6‐OHDA treatment. Synchronized late L4 larvae or young adults were transferred to fresh plates with or without RSV every other day (*n* = 50). The survival of the nematodes was monitored daily throughout the experiment. The survival rate on each plate was recorded until all the nematodes died. The lifespan experiments were repeated independently at least three times. Statistical analyses were performed using Kaplan–Meier lifespan analysis, and *P*‐values were calculated using the log‐rank test.

### 2.7 Quantification of DA Neurodegeneration, Fe^2+^, ROS, and Mitochondrial Damage in *C. elegans* Model

Quantitative analysis of DA neurodegeneration and mitochondrial damage was performed in transgenic BZ555 and PD4251 strains, respectively, under the treatment conditions described above. After 48 h of treatment, the worms were washed three times with M9 buffer and fixed onto an agar pad on a glass slide. They were then straightened using 100 mM sodium azide before being sealed with a coverslip. Fluorescence microscopy was used to assess DA neurodegeneration and mitochondrial damage in each group.

Quantitative analysis of Fe^2+^ and ROS levels was performed in N2 nematodes, with the same treatment and groups mentioned above. After 48 h of treatment, the nematodes were exposed to bacterial solutions containing 150 μM ROS probe (DCFH‐DA) or 10 μM Fe^2+^ probe (FerroOrange) for 2 h, following a 1‐h starvation period. ROS and Fe^2+^ levels were analyzed using a fluorescence microscope.

### 2.8 Experimental Design of Mice

Seventy mice were randomly assigned to control, PD, RSV10, RSV20, RSV30, ferrostatin‐1 (Fer‐1) and levodopa (Lev) groups (10 per group) (Figure 6A). A separate cohort of 50 mice was divided into control, PD, RSV, EX527, and ML385 groups (10 per group) (Figure 8A). Mice in the PD, RSV, Fer‐1, Lev, EX527, and ML385 groups received intraperitoneal injections of MPTP (30 mg/kg/day) for 5 consecutive days (days 1–5) [11]. From day 6 to day 15, the RSV group was administered RSV (10, 20 or 30 mg/kg/day) intraperitoneally, the Fer‐1 group was administered Fer‐1 (3 mg/kg/day) intraperitoneally, the Lev group was administered Lev (3 mg/kg/day) intraperitoneally. while the EX527 (5 mg/kg/day) and ML385 (30 mg/kg/day) groups were treated with the respective compounds 1 h prior to RSV (30 mg/kg/day) administration. The control group received an equivalent volume of 0.9% saline.

### 2.9 Detection of Iron Levels

Mouse brain tissues or cells were collected and homogenized, and the supernatants were obtained by centrifugation. Total iron levels were quantified using an iron assay kit, and the supernatant was analyzed for iron content using an ultraviolet spectrophotometer at 593 nm. For iron staining, Prussian blue staining was performed on 5 μm paraffin sections of three brain tissues. The sections were deparaffinized and rehydrated in distilled water, followed by sequential incubation with Perls' working solution, incubation solution, and enhanced working solution. After three washes with PBS, the sections were counterstained, dehydrated through a graded ethanol series, cleared in xylene, and sealed with resin.

### 2.10 Statistical Analysis

All data were analyzed using GraphPad Prism 8.0 software (San Diego, CA, USA). Data are expressed as the mean ± standard deviation (SD). The Shapiro–Wilk test was used for the normality test. Nonparametric tests were employed for data that did not follow a normal distribution. Comparison of means among groups was performed using the one‐way ANOVA followed by Tukey's post hoc test. A value of *p* < 0.05 was considered statistically significant.

## 3 Results

### 3.1 Iron Chelating Activity and Antioxidant Properties of RSV

**FIGURE 2:**
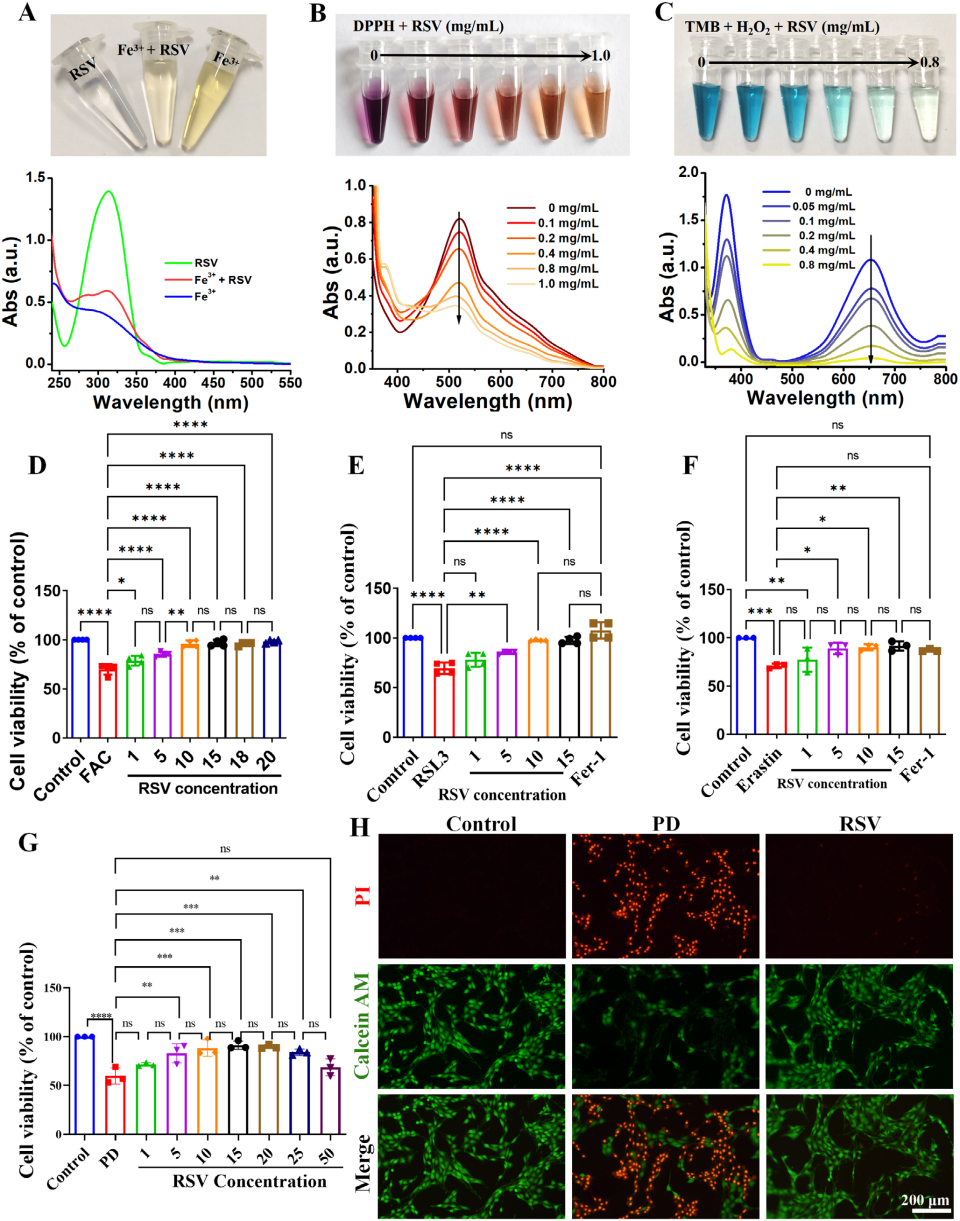
Iron‐chelating activity and antioxidant properties of RSV. (A) Chelation effect of RSV for Fe^3+^. (B–C) Antioxidant activity of RSV for DPPH assays and TMB‐H_2_O_2_ system. (D–F) The effect of RSV on the survival of cells induced by FAC, RSL3 and Erastin. (G) The effect of RSV on the survival of the PD model at different concentrations (1, 5, 10, 15, 20, 25 and 50 μM). (H) Calcein‐AM/PI staining in PC12 cells stimulated with 6‐OHDA and/or pretreated with RSV. *p < 0.05, **p < 0.01, ***p < 0.001, ****p < 0.0001, ns: no significant.

RSV is a plant‐derived polyphenolic compound with natural chelating activity, attributed to the catechol moieties in its structure, which contain three potential metal‐binding sites. Given the critical role of iron in ferroptosis, the iron‐chelating capacity of RSV was systematically evaluated by titrating RSV with trivalent iron (Fe^3+^) in a neutral buffer for UV–vis spectrophotometric analysis. With the addition of Fe^3+^, the characteristic absorbance of free RSV at 312 nm decreased, and the color of the Fe^3+^ solution became lighter, suggesting that RSV may chelate iron ions (Figure 2A). We further assessed the antioxidant capacity of RSV using the well‐established 1,1‐diphenyl‐2‐picrylhydrazide (DPPH) assays and 3,3′,5,5′‐tetramethylbenzidine (TMB)‐H_2_O_2_ system. Results demonstrated that RSV efficiently scavenged DPPH• radicals and H_2_O_2_ in a dose‐dependent manner, respectively (Figure 2B,C). These results indicate the potential of RSV in iron chelation and antioxidant activity.

Subsequently, the impact of RSV on neuronal survival under iron overload was assessed. The MTT assay was conducted to determine the optimal concentration of RSV for the experiment. High RSV concentrations (≥ 30 μM) were found to induce cytotoxicity. Consequently, 10 μM of RSV was selected as the optimal concentration for subsequent experiments (Figure S1). PC12 cells were treated with ferric ammonium citrate (FAC) to establish an in vitro model of iron overload‐induced neuronal injury, resulting in a rapid decline in cell viability to approximately 61.8% (Figure S2). RSV treatment significantly increased cell viability, and the cell survival rate was 98.6%, which was close to the control level. (Figure 2D). These results suggest that RSV may have protective effects against ferroptosis.

### 3.2 RSV Mitigates Ferroptosis of PC12 Cells Induced by Erastin‐ and RSL3

To further investigate RSV's anti‐ferroptotic effects, PC12 cells were treated with two classical ferroptosis inducers, Erastin and RSL3. As shown in Figure 2E,F, RSV pretreatment effectively attenuated cytotoxicity induced by both compounds. Comparative analysis with the ferroptosis inhibitor ferrostatin‐1 (Fer‐1) revealed that RSV exhibited superior cytoprotective effects in a dose‐dependent manner. These results support the hypothesis that RSV can protect PC12 cells from Erastin‐ and RSL3‐induced ferroptosis.

### 3.3 RSV Protected Against 6‐OHDA‐Induced Cell Death by Inhibiting Ferroptosis

Given its potential as an iron chelator and ROS scavenger, as well as its ability to protect against Erastin‐ and RSL3‐induced ferroptosis in PC12 cells, RSV was hypothesized to alleviate cytotoxicity in a PD model. Initially, PC12 cells were exposed to varying doses and durations of 6‐OHDA to establish an in vitro PD model [12]. The results revealed a dose‐dependent cytotoxic effect of 6‐OHDA, with concentrations ranging from 50 to 400 μM (Figure S3). At 200 μM, cell viability dropped to approximately 50% (50 ± 2.42, *p* < 0.0001). Consequently, 200 μM of 6‐OHDA was selected for subsequent experiments. PC12 cells were then treated with or without Fer‐1 (1 μM, 1 h), followed by 6‐OHDA. Fer‐1 treatment effectively mitigated 6‐OHDA‐induced cell death (Figure S4), consistent with previous studies suggesting that 6‐OHDA induces ferroptosis in PC12 cells [13]. To determine whether RSV could protect against 6‐OHDA‐induced ferroptosis, PC12 cells were treated with RSV (1–50 μM) prior to 6‐OHDA exposure. RSV at 15 μM provided the most significant protective effect, with cell viability reaching 90.22% ± 5.08% (Figure 2G). Calcein‐AM/propidium iodide (PI) cell staining was used to identify live and dead PC12 cells exposed to 6‐OHDA with or without RSV. Both the photographs and quantitative examination revealed that after 6‐OHDA stimulation, there was a prominent number of dead cells, whereas RSV treatment caused a noticeable increase in the number of viable cells (*p* < 0.05; Figure 2H and Figure S5). These results suggest that RSV may inhibit 6‐OHDA‐induced ferroptosis.

### 3.4 RSV Reduced Labile Iron Pool and LPO Levels in PD Cell Models by Chelating Iron and Removing ROS

**FIGURE 3:**
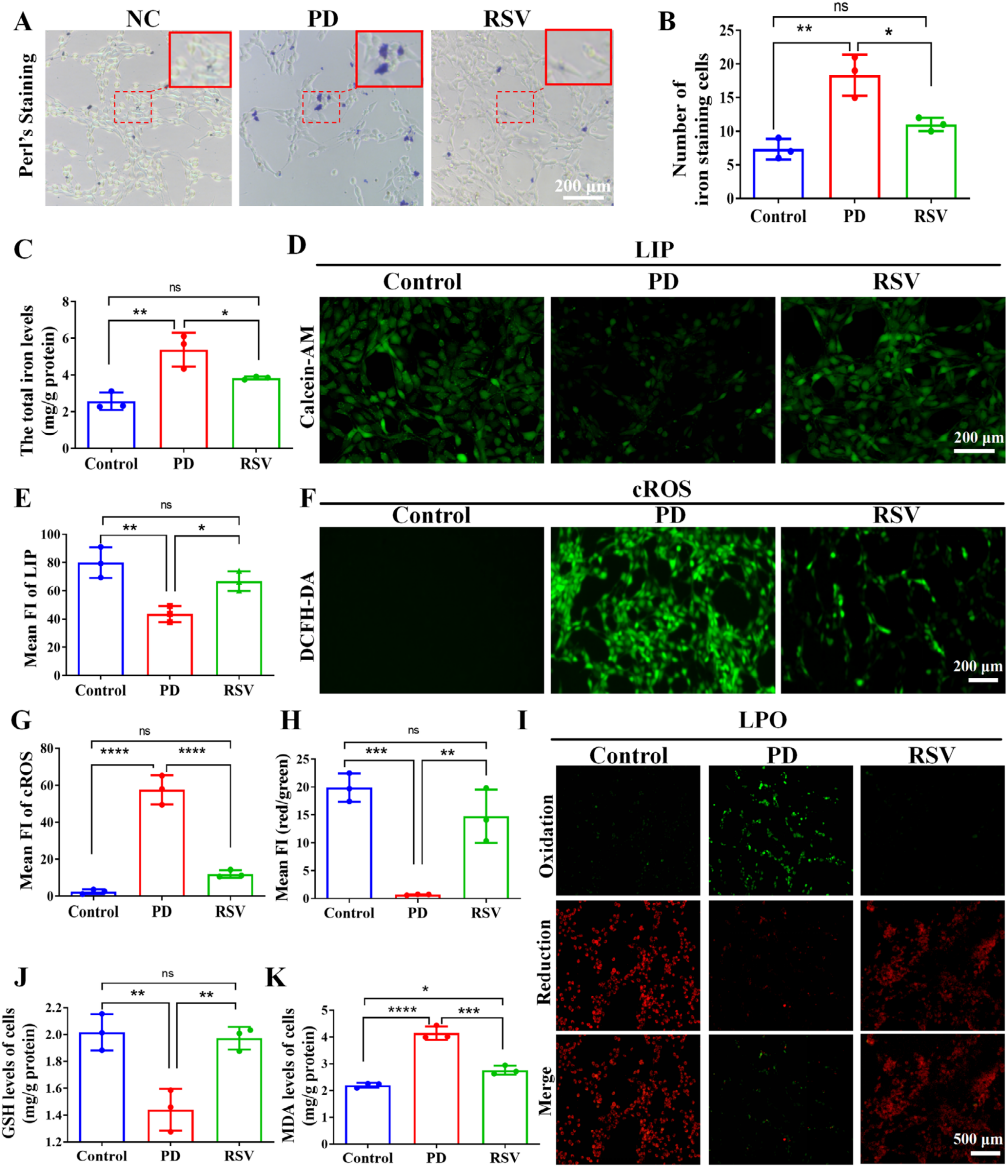
RSV reduced labile iron pool and LPO levels in PD cell models by chelating iron and removing ROS. (A–B) Perls' staining for visualization of the iron distribution in PC12 cells treated with or without RSV. (C) The levels of total iron in PC12 cells were measured using an iron assay kit. (D–E) Calcein‐AM dye was used to assess the LIP in PC12 cells treated with or without RSV. (F–G) DCFH‐DA analysis of the ROS levels in each group of cells. (H–I) Liperfluo staining for visualization of the LPO levels in each group of cells. (J–K) Quantitatively analyzed levels of intracellular GSH and MDA. Data expressed are as mean ± SD (*n* = 3 per group). *p < 0.05, **p < 0.01, ***p < 0.001, ****p < 0.0001.

Excess intracellular iron contributes directly to ferroptosis. To assess the effect of RSV on iron levels in PD cell models, Prussian blue staining was performed to detect iron‐positive cells, and total iron content was quantified. Prussian blue staining revealed a 3‐fold increase in iron‐positive cells in the PD group versus controls (*p* < 0.01), which was reduced by 50% following RSV treatment (*p* < 0.05; Figure 3A,B). Consistent with these findings, quantitative analysis demonstrated significantly elevated total iron content in PD cells (*p* < 0.01), with RSV treatment effectively normalizing iron levels (*p* < 0.05) (Figure 3C). Further, Calcein‐AM dye was used to assess the intracellular labile iron pool (LIP) [14]. Fluorescence imaging revealed quenched green fluorescence, indicating a marked increase in the LIP, suggesting that 6‐OHDA pretreatment augmented the intracellular LIP (*p* < 0.01). Upon treatment with RSV, the fluorescence intensity increased (*p* < 0.05; Figure 3D,E), indicating a continuous reduction in the intracellular LIP. These results collectively support that RSV's cellular actions involve iron chelation.

LPO, a pivotal process in ferroptosis, leads to excessive ROS generation. To assess the effect of RSV on ROS levels, ROS content was measured in each group. Quantitative analysis revealed that 6‐OHDA treatment induced a dramatic elevation in intracellular ROS levels compared to normal controls (*p* < 0.0001), while RSV treatment significantly reduced ROS levels in the PD model group (*p* < 0.0001; Figure 3F,G). This suggests that RSV can attenuate ROS accumulation, a central product of LPO, in PD cell models. Using a LPO fluorescent probe, we further confirmed that RSV significantly suppressed 6‐OHDA‐induced lipid peroxidation (*p* < 0.01; Figure 3H,I).

To further evaluate LPO, we quantified the levels of GSH and MDA, two critical biomarkers of oxidative stress. GSH mitigates oxidative stress in ferroptotic cells, while MDA is a byproduct of free radicals and LPO. Our findings revealed a marked depletion of GSH in PD model cells relative to normal controls (*p* < 0.01), which was substantially reversed by RSV administration (*p* < 0.01; Figure 3J). In contrast, we observed a pronounced accumulation of MDA in PD cells relative to normal controls (*p* < 0.0001), but RSV treatment significantly attenuated MDA production (*p* < 0.001; Figure 3K). In addition, we observed a significant increase in 4‐HNE in PD cells compared to normal controls (*p* < 0.0001), but RSV treatment remarkably reduced the production of 4‐HNE (*p* < 0.0001; Figure S6).

### 3.5 RSV Protected Against 6‐OHDA‐Induced Mitochondrial Dysfunction in PC12 Cells

**FIGURE 4:**
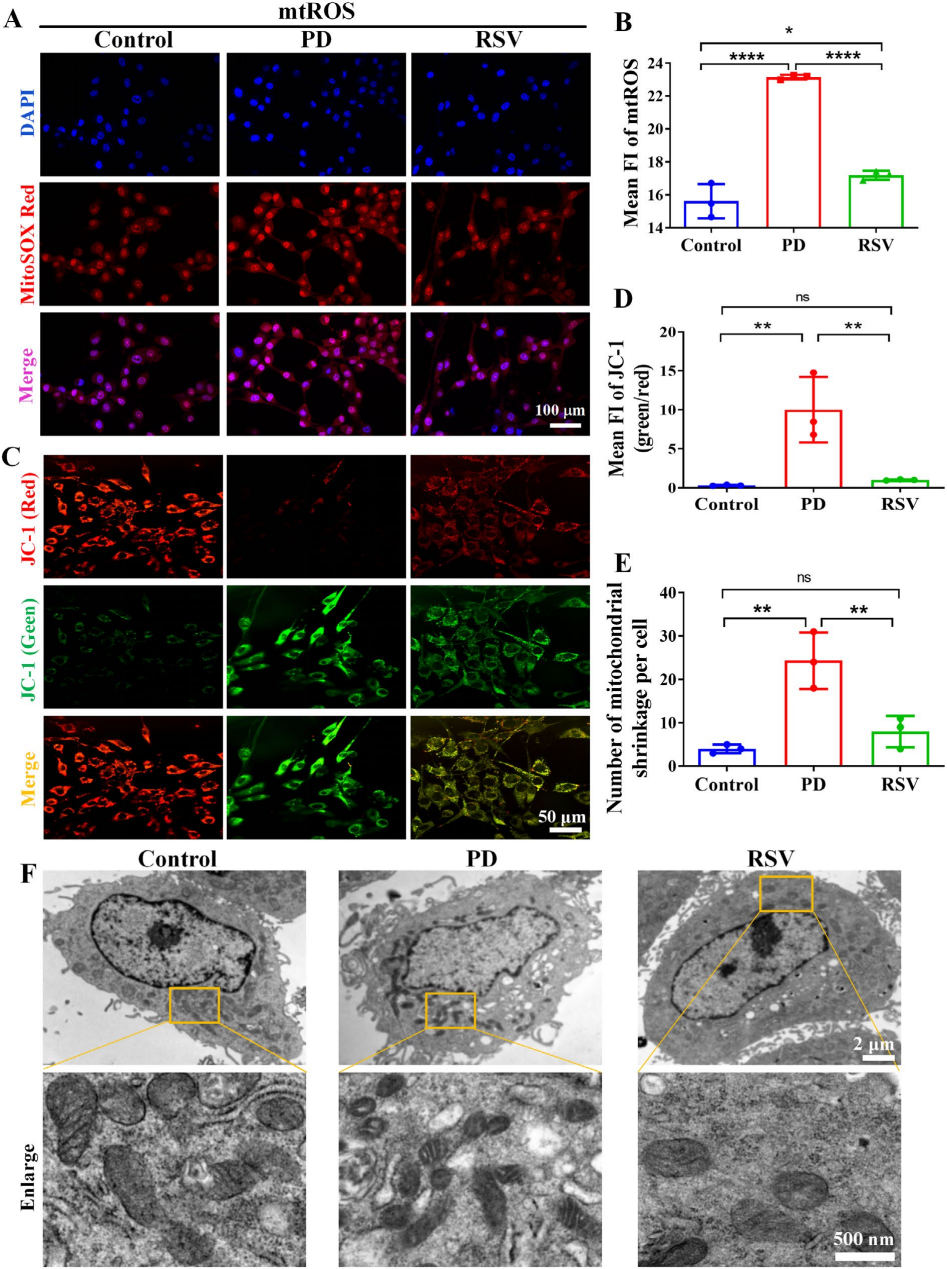
RSV Protected against 6‐OHDA‐induced mitochondrial dysfunction in PC12 cells. (A–B) MitoSOX analysis of the mROS levels in each group of cells. (C–D) JC‐1 staining was performed to assess mitochondrial damage by measuring the ratio of green to red fluorescence. (E–F) Electron micrographs showed mitochondrial morphology changes associated with ferroptosis in the PD group, and RSV rescued these changes. (*n* = 3 per group). *p < 0.05, **p < 0.01, ****p < 0.0001, ns: No significant.

Mitochondria are primary sources of ROS and are closely linked to ferroptosis. To systematically evaluate mitochondrial functional alterations induced by 6‐OHDA exposure and the potential protective effects of RSV, we quantitatively assessed mitochondrial ROS (mROS) production using MitoSOX Red fluorescence staining. Comparative analysis revealed a marked increase in mROS in the PD group compared to the NC group (*p* < 0.0001). However, RSV treatment significantly attenuated mROS levels, highlighting its capacity to scavenge mROS and mitigate oxidative stress‐induced damage (*p* < 0.0001; Figure 4A,B).

The JC‐1 probe, a widely used fluorescent marker for detecting mitochondrial membrane potential (Δψm), was employed to assess Δψm alterations, a key indicator of ferroptosis. The probe accumulates in mitochondria in a Δψm‐dependent manner, with changes in staining reflecting mitochondrial polarization. The ratio of green to red fluorescence was calculated to quantify mitochondrial depolarization. Compared to the NC group, the ratio of green to red fluorescence in the PD group was significantly increased (*p* < 0.01). RSV treatment significantly reduced this ratio (*p* < 0.01), suggesting that RSV alleviated mitochondrial depolarization in the PD model (Figure 4C,D).

The primary morphological characteristics of mitochondria undergoing ferroptosis include increased mitochondrial membrane density, reduced volume, and the loss of inner cristae structure. To evaluate the impact of RSV on these morphological changes, transmission electron microscopy was used to examine mitochondrial ultrastructure in each group. The results revealed that, compared to the NC group, the PD group exhibited typical ferroptotic mitochondrial alterations, such as increased membrane density, decreased volume, and the disappearance of inner cristae. Notably, RSV treatment significantly ameliorated these morphological changes (*p* < 0.01; Figure 4E,F). Taken together, these results suggest that RSV improves mitochondrial function and mitigates the detrimental effects of 6‐OHDA treatment.

### 3.6 RSV Reduce Iron and ROS Accumulation to Exert Neuroprotective Effects in a PD Nematode Model

Numerous studies have established a strong correlation between iron accumulation, ROS production, and oxidative stress, all of which contribute to mitochondrial damage and ultimately lead to the death of DA neurons. To further investigate the neuroprotective potential of RSV across different species, a *Caenorhabditis elegans* ( *C. elegans* ) model was employed to observe the effects on iron levels, ROS production, and DA neuron morphology.

**FIGURE 5:**
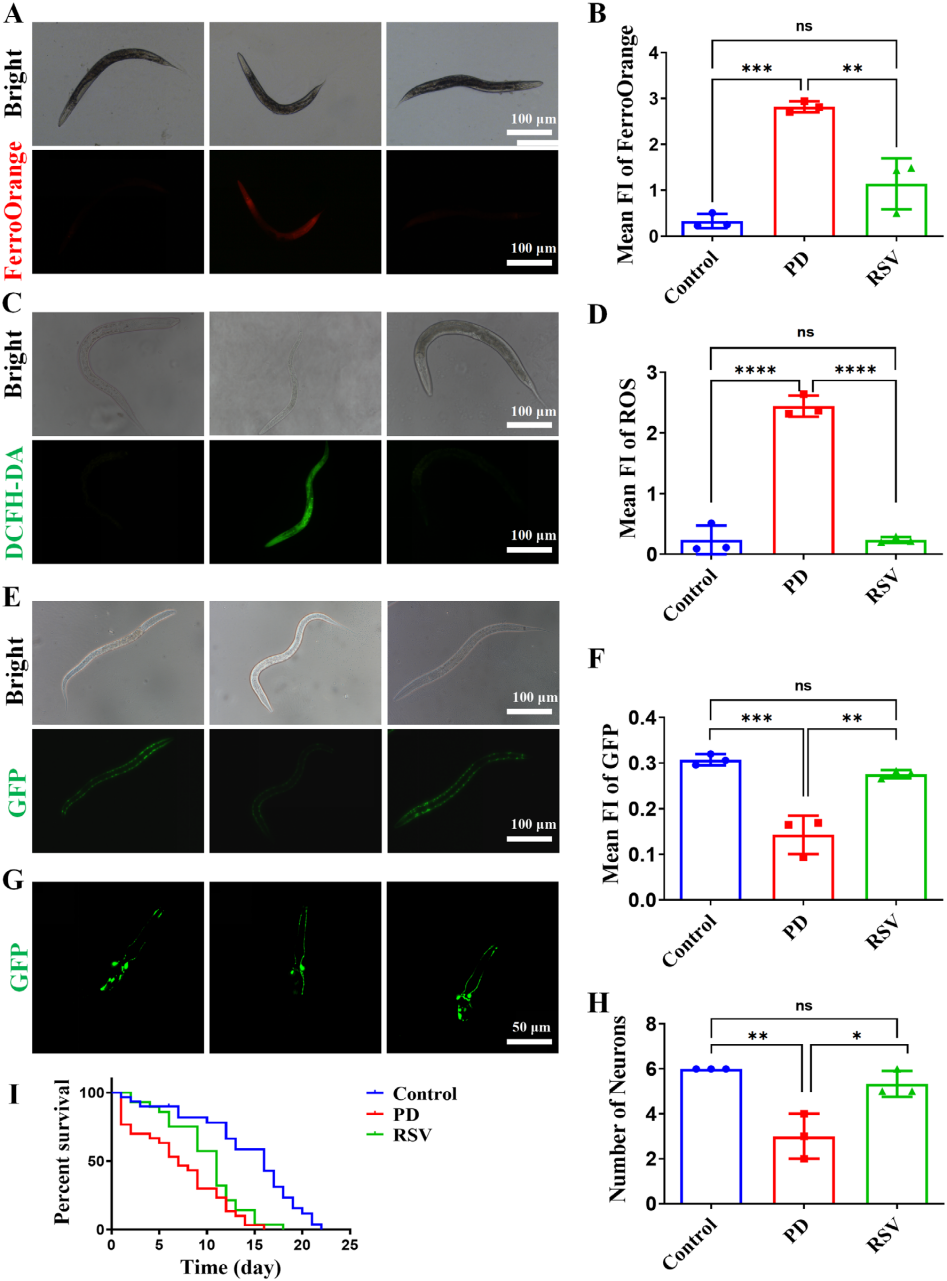
RSV reduces iron and ROS accumulation to exert neuroprotective effects in a PD nematode model. (A–B) FerroOrange staining of N2 nematodes stimulated with 6‐OHDA and pretreated with RSV. (C–D) DCFH‐DA analysis of the ROS levels in each group of nematodes. (E–F) Fluorescence images of mitochondria in PD4251 nematodes stimulated with 6‐OHDA and pretreated with RSV. (G–H) Fluorescence images of DA neurons in BZ555 nematodes stimulated with 6‐OHDA and pretreated with RSV. (I) Survival curve of N2 worms grown in the presence of 6‐OHDA and RSV. Data expressed are as mean ± SD (*n* = 3 per group). *p < 0.05, **p < 0.01, ***p < 0.001, ****p < 0.0001, ns: No significant.

The 6‐OHDA‐pretreated wild‐type *C. elegans* (Bristol N2 strain, did not have any markers) was initially utilized to assess Fe^2+^ and ROS levels [15, 16]. As depicted in Figure 5A,B, red FerroOrange (a detection probe for Fe^2+^) fluorescence was prominently observed in the nematodes of the PD group (*p* < 0.001). In contrast, RSV treatment resulted in a noticeable reduction in red fluorescence intensity, indicating a marked decrease in Fe^2+^ production compared to the PD group (*p* < 0.01). This suggests that RSV effectively attenuates iron accumulation in the model. Subsequently, the 6‐OHDA‐pretreated Bristol N2 strain model was used to evaluate ROS levels. The DCFH‐DA assay revealed a relative increase in intracellular ROS levels in the PD group compared with that in the control group (*p* < 0.0001). However, RSV treatment significantly reduced the green fluorescence intensity (*p* < 0.0001; Figure 5C,D), further validating its role in reducing Fe^2+^ levels and eliminating ROS.

Mitochondrial damage was assessed using the PD4251 transgenic nematode model, which expresses GFP in body wall muscle mitochondria [17]. As illustrated in Figure 5E,F, the fluorescence intensity was significantly reduced in the PD group (*p* < 0.001), indicating mitochondrial damage. Remarkably, RSV treatment effectively restored fluorescence levels (*p* < 0.01), suggesting a protective role in mitigating 6‐OHDA‐induced mitochondrial dysfunction.

To further evaluate the neuroprotective effects of RSV, we employed the BZ555 nematode strain, a PD model featuring GFP‐labeled DA neurons (DAns) [18]. 6‐OHDA treatment significantly reduced the number of DAns in the head region (*p* < 0.01), indicating substantial neuronal damage. Notably, RSV treatment restored DAn integrity (*p* < 0.05; Figure 5G,H), supporting its potential to protect DA neurons from degeneration. Finally, we assessed the impact of RSV on lifespan using 6‐OHDA‐treated Bristol N2 nematodes. The median survival time (MST) of the 6‐OHDA‐treated nematodes was markedly reduced (7 days) compared to controls (16 days). Strikingly, RSV treatment extended the MST to 11 days (Figure 5I), further corroborating its neuroprotective efficacy in this PD model.

### 3.7 RSV Ameliorates Motor Deficits and Restored DA Neurodegeneration in SN of Mice Induced by MPTP

**FIGURE 6:**
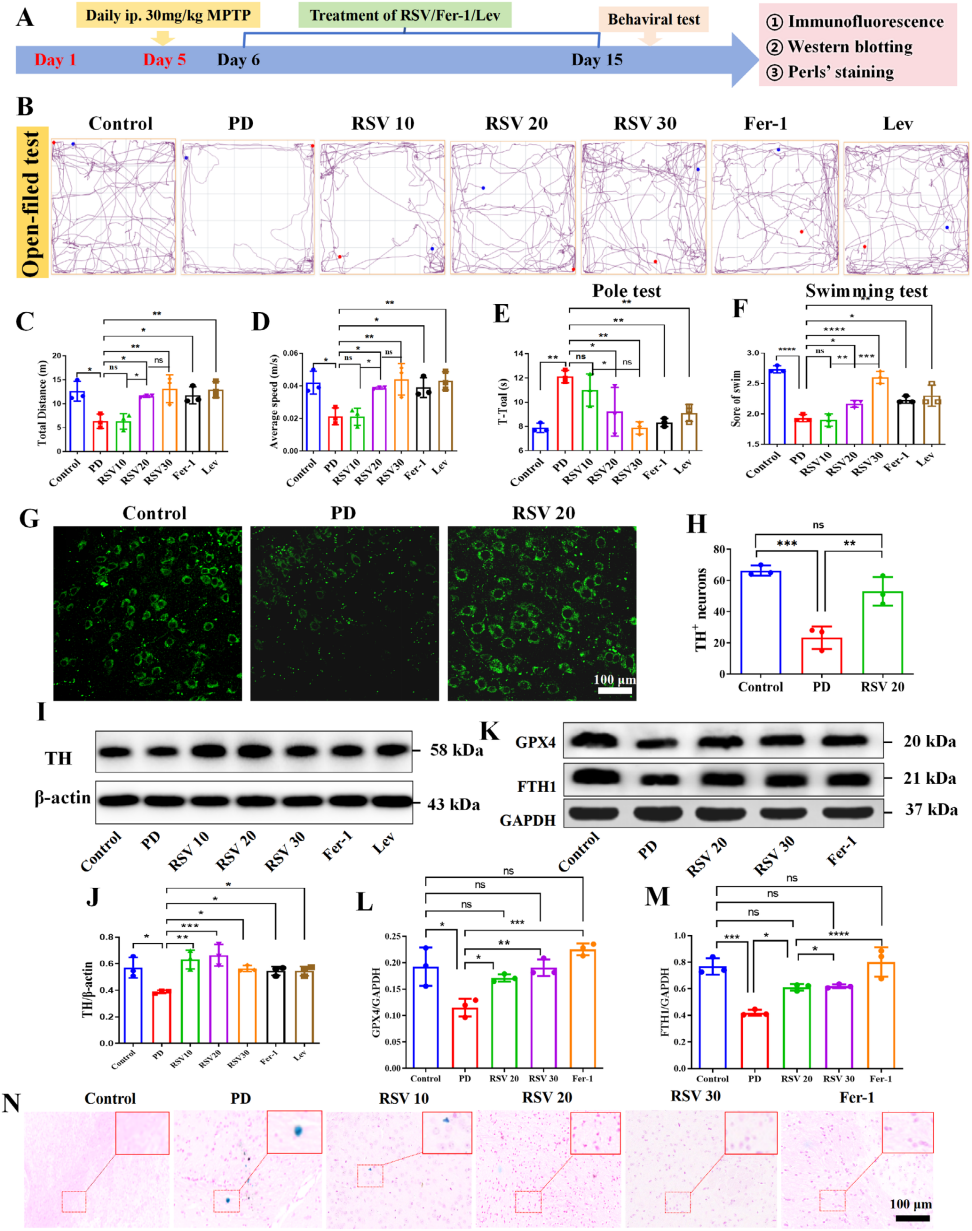
(A) Schematic overview of the comprehensive experimental procedure for RSV/Fer‐1/Lev treatment of MPTP‐induced PD. The behavior assessment included various tests, including the Open Field Test (B–D), Pole‐Climbing Test (E), and Forced Swimming Test (F). (G–H) Representative images and quantification of TH‐positive staining cells in the SN of mice. (I–J) Western blot analysis results of TH levels in the SN of mice. (K–M) Western blot analysis results of GPX4 and FTH1 levels in the SN of mice. (N) Iron‐staining cells in the SN of mice using Perl's Stain Kit. Data expressed as mean ± SD (*n* = 3 per group). *p < 0.05, **p < 0.01, ***p < 0.001, ****p < 0.0001, ns: No significant.

To evaluate whether RSV alleviates motor deficits in a mouse model of PD, various behavioral assays, including forced swimming, open field, and pole climbing tests, were conducted. In the open field test, RSV‐treated groups (20 and 30 mg/kg/day) exhibited significant improvements in locomotor activity, with both total movement distance and average speed showing marked increases compared to the PD model group (Figure 6B–D). Similar enhancements were observed in Fer‐1 and Lev treatment groups. The pole climbing test revealed substantial motor coordination improvements across all treatment groups. RSV administration (20 and 30 mg/kg) reduced climbing time by 23.8% and 34.9%, respectively, comparable to the effects of Fer‐1 (31.2% reduction) and Lev (24.8% reduction) (Figure 6E). Consistent with these findings, the forced swimming test indicated significantly reduced immobility scores in treated groups (RSV20:2.17 ± 0.058, RSV30: 2.60 ± 0.10, Fer‐1: 2.23 ± 0.58, and Lev:2.30 ± 0.17) versus the PD model group (1.93 ± 0.58) (Figure 6F). Collectively, these behavioral assessments demonstrate that both RSV doses (20 and 30 mg/kg/day) effectively ameliorate MPTP‐induced motor impairments in mice, with efficacy comparable to that of Fer‐1 and Lev treatments.

A key pathological hallmark of PD is the degeneration of DA neurons. Tyrosine hydroxylase (TH), an enzyme critical to DA synthesis, serves as a marker for DA neuron damage [19]. To assess the extent of DA neuron loss in the SN, the number of TH‐positive neurons and the expression of TH protein were evaluated. The results demonstrated that RSV treatment (20 mg/kg/day) significantly restored the number of TH‐positive neurons compared to the PD model group (*p* < 0.01; Figure 6G,H). Furthermore, Western blot analysis revealed a marked upregulation of TH protein expression in all treatment groups relative to the PD model group (*p* < 0.05; Figure 6I,J). These findings suggest that RSV effectively attenuates MPTP‐induced neurodegeneration of DA neurons in the SN, with a neuroprotective efficacy comparable to that of Fer‐1 and Lev treatments.

### 3.8 RSV Regulated Ferroptosis‐Associated Proteins and Decreased Iron Levels in PD Mice

The Xc‐system‐GSH‐GPX4 axis is a well‐known regulator of ferroptosis, with GPX4 playing a pivotal role in clearing lipid peroxides, thus acting as a key negative regulator of ferroptosis [20]. Ferritinophagy, regulated by FTH1, also inhibits ferroptosis [21]. The expression levels of GPX4 and FTH1 in the SN region of the mice were examined. The results revealed that both RSV doses (20 and 30 mg/kg/day) as well as Fer‐1 treatment significantly enhanced the expression levels of GPX4 and FTH1 compared to the PD model group (*p* < 0.05, *p* < 0.05; Figure 6K–M), suggesting that RSV may protect against ferroptosis through this axis.

Prussian blue staining was used to examine iron deposition in the SN region. The results demonstrated significantly elevated iron deposition in the SN of PD mice compared to the NC group. Notably, both RSV treatment (20 and 30 mg/kg/day) and Fer‐1 administration effectively attenuated this iron accumulation, as evidenced by reduced iron‐positive cell counts (Figure 6N). These results suggest that RSV possesses iron‐chelating properties comparable to Fer‐1, potentially contributing to its neuroprotective effects in this PD model.

### 3.9 RSV Target Prediction in the Treatment of PD

**FIGURE 7:**
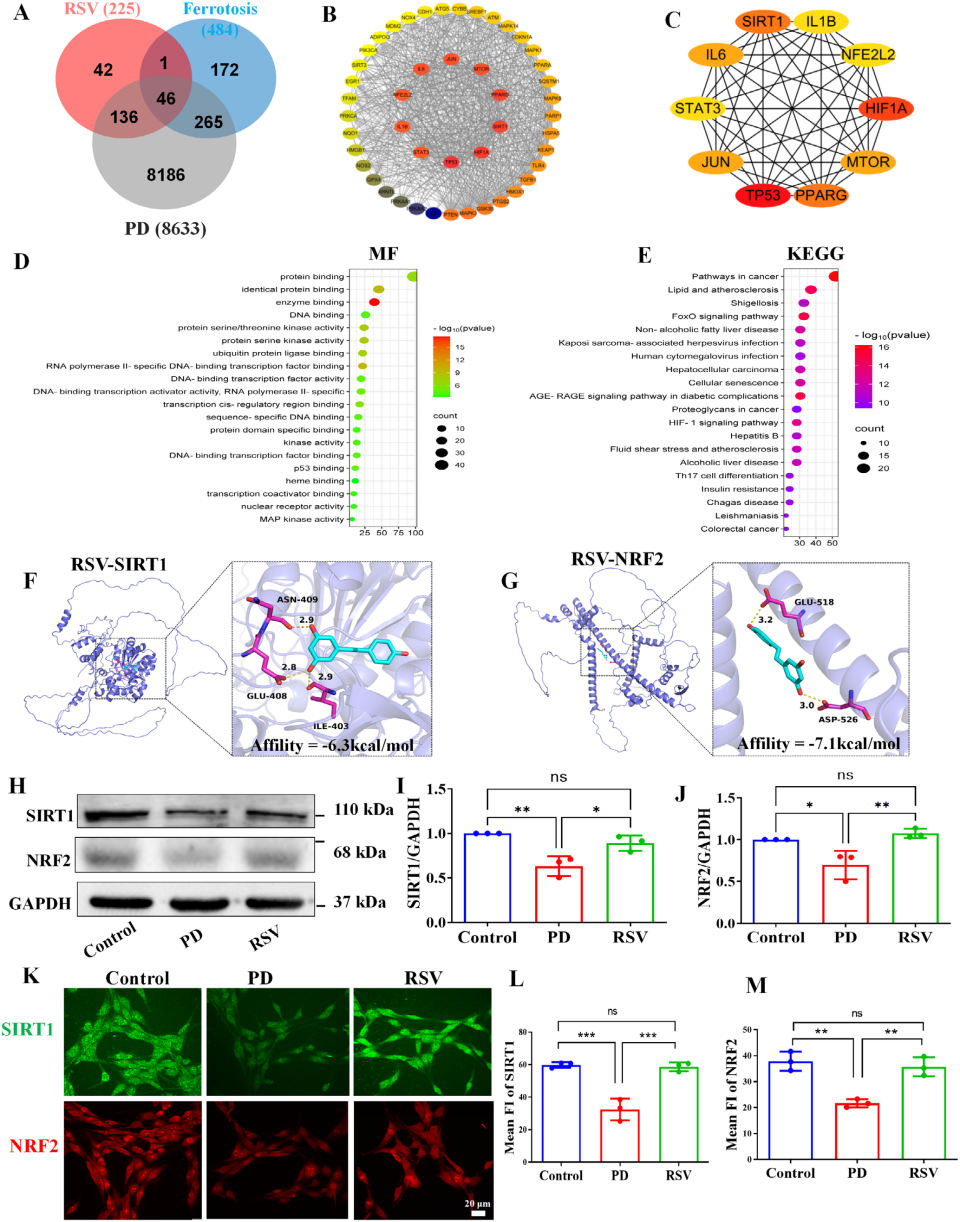
(A) Venn diagrams of potential targets of RSV, PD and ferroptosis. (B) Core gene screening in the PPI network. The shade of color represents the degree of change. (C) The top 10 targets based on the value of Degree. (D) GO enrichment Molecular function (MF). (E) KEGG enrichment pathway. (F) Molecular docking between RSV and SIRT1. (G) Molecular docking between RSV and NRF2. (H–J) Western blot and quantification of SIRT1 and NRF2 levels in the SN of mice. (K–M) Immunofluorescence and quantification of SIRT1 and NRF2 levels in PC12 cells. The results of three independent experiments are represented as mean ± S.D. *n* = 3. *p < 0.05, **p < 0.01, ***p < 0.001, ns: No significant.

To identify potential therapeutic targets of RSV for PD and ferroptosis, 225 RSV‐related targets, 484 ferroptosis‐related targets, and 8633 PD‐related targets were retrieved from existing datasets. The Venn diagram revealed 46 overlapping targets among RSV, ferroptosis, and PD, which were considered potential therapeutic targets (Figure 7A). These targets were further analyzed using the STRING database to evaluate protein–protein interactions, and the resulting data were visualized using Cytoscape 3.9.0 (Figure 7B). The top ten core targets, identified based on their degree values, included SIRT1, TP53, HIF1A, PPARG, MTOR, JUN, IL6, IL1B, STAT3, and NFE2L2 (Figure 7C).

### 3.10 Pathway Enrichment Study Using GO and KEGG

To explore the biological processes and signaling pathways associated with the overlapping targets, GO and KEGG enrichment analyses were performed. As shown in Figure S7A, the BP analysis revealed that the target genes were primarily enriched in processes such as the positive regulation of RNA polymerase II, positive regulation of gene expression, cellular response to oxidative stress, and negative regulation of ferroptosis. In the CC analysis (Figure S7B), enrichment was observed in the nucleus, cytosol, cytoplasm, nucleoplasm, and mitochondrion. In terms of MF (Figure 7D), protein binding, identical protein binding, and enzyme binding were identified as key molecular activities. KEGG analysis (Figure 7E) highlighted the involvement of the FoxO signaling pathway and cellular senescence in the regulation of these targets. Notably, silent information regulator 1 (SIRT1) emerged as a central signal in these pathways, prompting further focus on its role in RSV's therapeutic effects on PD.

### 3.11 RSV Up‐Regulated SIRT1‐NRF2 Signaling Pathway in PD Mice Model

To validate the network pharmacology findings regarding the interaction between key targets and RSV, molecular docking was performed to investigate the binding affinity of RSV with SIRT1 and NRF2, two critical proteins identified in the analysis. The docking results demonstrated robust and stable binding interactions between RSV and both target proteins. Specifically, RSV formed multiple hydrogen bonds with SIRT1 at critical residues, including ILE403, GLU408, and ASN409, exhibiting a binding affinity of −6.3 kcal/mol (Figure 7F and Table S1). Similarly, RSV exhibited strong binding with NRF2, engaging residues GLU518 and ASP526, and yielding a favorable binding energy of −7.1 kcal/mol (Figure 7G and Table S2). These interactions confirm the potential of RSV to bind to these key proteins.

To further validate the role of the SIRT1‐NRF2 pathway in RSV's figure using WB analysis. The results revealed a significant upregulation of both SIRT1 and NRF2 in the RSV‐treated group compared to the PD model group (Figure 7H–J). To further validate our molecular docking predictions, we conducted in vitro experiments, which confirmed that RSV treatment markedly enhanced the protein expression of both SIRT1 and NRF2 (*p* < 0.05, *p* < 0.01; Figure 7K–M). These results are consistent with the results from the network pharmacology analysis and molecular docking.

### 3.12 RSV Ameliorates Motor Deficits and Ferroptosis by Upregulating the SIRT1/NRF2 Pathway

**FIGURE 8:**
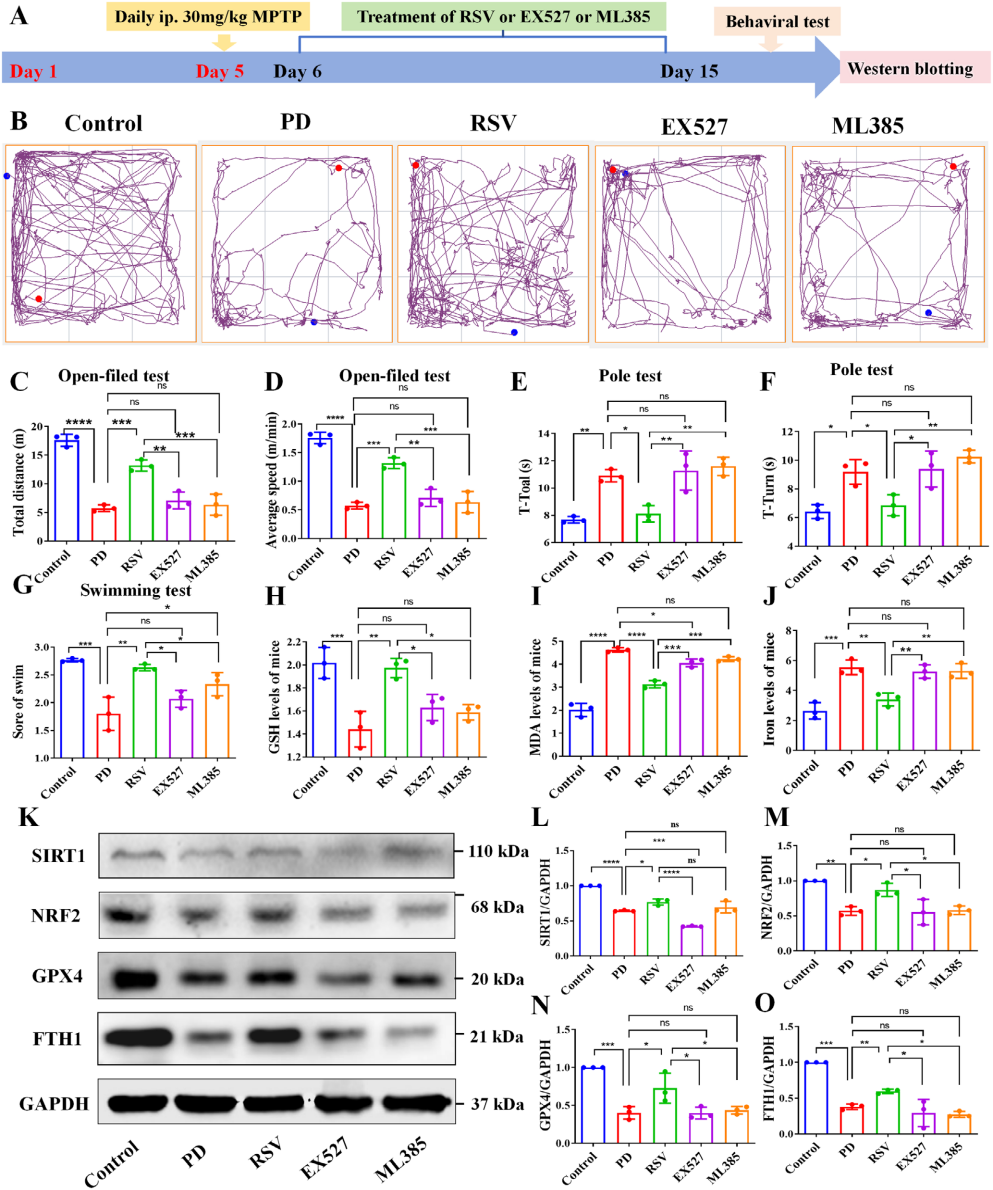
RSV ameliorates motor deficits and ferroptosis by upregulating the SIRT1/NRF2 pathway. (A) Schematic overview of the comprehensive experimental procedure. The behavior assessment included various tests, including the Open field test (B–D), Pole‐climbing test (E–F), and Forced swimming test (G). (H–J) Quantitatively analyzed levels of intracellular GSH, MDA and iron. (K–O) Western blot analysis results of SIRT1, NRF2, GPX4 and FTH1 levels in the SN of mice. Data expressed as mean ± SD (*n* = 3 per group). *p < 0.05, **p < 0.01, ***p < 0.001, ****p < 0.0001, ns: Not significant.

To investigate whether the effects of RSV on motor deficits in the PD mouse model are linked to the SIRT1‐NRF2 signaling pathway, this study employed pharmacological inhibition of SIRT1 and NRF2 using EX527 and ML385, respectively [22]. Motor function was evaluated through open field, pole climbing, and forced swimming experiments. The open field experiment revealed that inhibition of SIRT1 significantly reduced the total distance and average speed of mouse movement compared to the RSV treatment group (*p* < 0.01; Figure 8B–D). The same phenomenon was detected after inhibition of NRF2 compared to the RSV treatment group (*p* < 0.001). Furthermore, the pole climbing time was notably increased in the inhibitor‐treated groups compared to the RSV‐treated mice (*p* < 0.05; Figure 8E,F). Similarly, in the forced swimming test, swim scores were significantly higher in the SIRT1 and NRF2 inhibitor groups than in the RSV‐treated group (*p* < 0.05, *p* < 0.05; Figure 8G). These results suggest that blocking SIRT1 and NRF2 effectively eliminated RSV's beneficial effects on MPTP‐induced motor deficits.

To further explore whether the effect of RSV on ferroptosis is mediated by the SIRT1/NRF2 pathway, key biomarkers involved in ferroptosis were assessed. The results demonstrated that inhibition of SIRT1 or NRF2 blocked the increase in GSH levels induced by RSV in PD mice (*p* < 0.05, *p* < 0.05; Figure 8H). The inhibitory effects of EX527 and ML385 on RSV were also evident in the decreased levels of MDA and total iron (*p* < 0.001, *p* < 0.001; Figure 8I,J). Moreover, SIRT1 and NRF2 inhibitors blocked the decrease in 4‐HNE levels induced by RSV in PD mice (*p* < 0.05, *p* < 0.05; Figure S8). Additionally, the expression of key proteins associated with the SIRT1–NRF2 pathway was examined. The results showed that EX527 treatment led to decreased expression of SIRT1, NRF2, FTH1, and GPX4 compared to the RSV group. In contrast, ML385 treatment resulted in reduced levels of NRF2, FTH1, and GPX4, but had no significant effect on SIRT1 expression compared to the RSV group (Figure 8K–O and Figure S9).

## 4 Discussion

The primary pathological features of PD involve the progressive degeneration and death of DA neurons in the SN region [23]. Apoptosis is associated with extensive degeneration of DA neurons in PD patients [24]. However, apoptosis inhibitors have shown little efficacy in PD patients in clinical trials, suggesting that apoptosis is not a major mechanism in PD. In contrast, a recent study suggested that ferroptosis may occur in the early stages of PD prior to apoptosis [25]. A hallmark of ferroptosis is elevated levels of lipid peroxides [26]. Combining ferroptosis inhibitors may offer a promising therapeutic strategy for PD, suggesting that targeting drugs to reduce iron accumulation in the brain could become a critical avenue for PD treatment [14, 27, 28].

Behavioral assessments, including open field, pole climbing, and forced swimming tests, are commonly employed to evaluate PD progression and therapeutic interventions. This study demonstrates that RSV alleviates MPTP‐induced behavioral impairments in mice across these tests, indicating its potential therapeutic effects. Given that the progressive degeneration of DA neurons is central to PD pathology, further investigation of DA neuronal degeneration is essential for assessing therapeutic efficacy [23]. TH, a key enzyme in DA synthesis, plays a critical role in the production of DA. Dysfunction or insufficient expression of TH directly impairs DA synthesis and secretion. Thus, TH levels serve as an indirect marker of DA neuronal integrity. The current study suggests that RSV can mitigate DA neuronal loss in a PD model, offering therapeutic benefits, though the precise underlying mechanism remains unclear.

Ferroptosis, a novel form of cell death driven by iron‐dependent ROS‐mediated LPO, is regulated by mechanisms involving iron and lipid metabolism, as well as the xCT/GPX4 pathway [29]. While studies have established that RSV modulates ferroptosis in the context of various diseases [9, 10], its role in regulating ferroptosis in PD remains uncertain.

Cellular iron accumulation is a hallmark of ferroptosis, with free iron being a key factor in promoting this form of cell death [30]. This study found that RSV effectively reduced both the number of iron‐positive cells and the total iron content in a PD cell model, as well as decreased the number of iron‐positive cells in PD mice. These findings suggest that the iron‐reducing properties of RSV may ameliorate ferroptosis, prompting further investigation into relevant indicators of iron‐mediated cell death to confirm RSV's anti‐ferroptotic effects.

ROS play a pivotal role in ferroptosis, where excessive ROS production is a central outcome of LPO, with MDA serving as a key marker of oxidative stress. In the PD model, iron accumulation can drive the generation of ROS through the Fenton reaction, leading to mitochondrial dysfunction and increased lipid ROS, a hallmark of ferroptosis. When GSH levels decrease, coupled with excessive MDA and iron accumulation, ferroptosis is triggered. The present study demonstrated that RSV inhibits MDA formation and ROS accumulation in the PD model, reduces LPO, and enhances GSH levels, thus mitigating ferroptosis.

GSH, synthesized from glutamic acid and cysteine, is critical for cellular protection, with GPX4 utilizing GSH to convert toxic lipid peroxides into non‐toxic lipid alcohols, thereby preserving membrane lipid bilayer integrity [31]. Ferroptosis can result in mitochondrial hyperpolarization [32], and assessing mitochondrial function and morphology is essential for evaluating ferroptosis [33, 34]. Preserving mitochondrial integrity is considered a key strategy to prevent ferroptosis. In this study, the PD group exhibited mitochondrial morphological alterations indicative of ferroptosis, including reduced size, increased membrane density, and the loss of internal ridge structure. RSV treatment significantly restored mitochondrial morphology in the PD cell model and reduced mitochondrial depolarization, improving mitochondrial function. The inactivation of GPX4 or depletion of GSH leads to lipid peroxide accumulation, contributing to ferroptosis [31]. Ferritinophagy, a selective form of autophagy involving the degradation of ferritin, has been linked to ferroptosis through the involvement of FTH1 [35]. This study revealed that RSV inhibited ferroptosis by upregulating GPX4 and FTH1 expression in the PD model. Thus, RSV mitigates ferroptosis in PD models, although the precise underlying mechanism remains unclear.

SIRT1, a nicotinamide adenine dinucleotide (NAD^+^)‐dependent deacetylase, regulates gene expression and cellular function by deacetylating various target proteins [36]. SIRT1 activity is closely linked to the cellular stress response and ferroptosis [37, 38]. Under oxidative stress, SIRT1 modulates the activity of transcription factors and coactivators through deacetylation, thereby regulating the expression of antioxidant genes. Studies have shown that SIRT1 directly deacetylates NRF2, enhancing its stability and transcriptional activity, which in turn boosts cellular antioxidant capacity. NRF2 regulates the expression of ferroptosis‐related proteins [39, 40]. Additionally, SIRT1 influences NRF2 activity, suggesting that the SIRT1/NRF2 signaling pathway could be a potential molecular mechanism through which RSV regulates ferroptosis in PD.

In this study, expression levels of SIRT1 and NRF2 were found to be downregulated in PD mice. RSV treatment upregulated both SIRT1 and NRF2, significantly reducing LPO levels. RSV alleviated ferroptosis in PD by upregulating FTH1 and GPX4. Inhibition of SIRT1 or NRF2 blocked the beneficial effects of RSV on motor deficits in PD mice. Following SIRT1 inhibition, levels of NRF2, GPX4, FTH1, and SIRT1 were significantly reduced. Similarly, NRF2 inhibition reduced GPX4 and FTH1 levels and exacerbated ferroptosis. These findings indicate that the SIRT1/NRF2 pathway plays a pivotal role in RSV‐mediated inhibition of ferroptosis in PD.

## 5 Conclusion

In summary, this study identifies potential therapeutic targets for RSV in mitigating ferroptosis in PD, utilizing an extensive approach that includes network pharmacology, bioinformatics analysis, and molecular docking simulations, supported by both in vivo and in vitro experiments. RSV treatment effectively modulates ROS and Fe^2+^ levels, reduces LPO and oxidative stress, upregulates GPX4 and FTH1 expression, improves mitochondrial function, and inhibits ferroptosis in PD. The underlying mechanism is likely linked to the SIRT1/NRF2 signaling pathway. In the future, exploring the role of other potential signaling pathways in conjunction with the SIRT1/NRF2 pathway may provide a more comprehensive understanding of how RSV influences ferroptosis.

## Ethics Statement

The animal study was reviewed and approved by the Ethical Committee of Guizhou Medical University (2304734).

## Conflicts of Interest

The authors declare no conflicts of interest.

## Supporting information


**Table S1:** Molecular docking between RSV and SIRT1.
**Table S2:** Molecular docking between RSV and NRF2.
**Figure S1:** The effect of RSV on the survival of PC12 cell at different concentrations.
**Figure S2:** The toxic effect of FAC at different concentrations.
**Figure S3:** The toxic effect of 6‐OHDA at different concentrations.
**Figure S4:** The effect of Fer‐1 on the survival of PD model at different concentrations.
**Figure S5:** Fluorescence quantitative analysis the images from Fig.2H.
**Figure S6:** The effect of RSV on 4‐HNE level in PD cell model.
**Figure S7:** GO enrichment analysis.
**Figure S8:** SIRT1 and NRF2 inhibitors blocked the decrease in 4‐HNE levels induced by RSV in PD mice model.
**Figure S9:** The effect of RSV on SIRT1‐NRF2 signal pathway.

## Data Availability

The data used to support the findings of this study are available from the corresponding author upon request. Supporting Information associated with this article can be found in the online version.
