# Supplementary material for: Groundbreaking Insights Into SIRT1/NRF2‐Mediated Ferroptosis Inhibition by Resveratrol in Parkinson's Disease Models
Source: CNS Neurosci Ther. 2025 Nov 11;31(11):e70648. doi: 10.1111/cns.70648 (PMC12605972; doi:10.1111/cns.70648)
Supplement: Supplementary file 1 — Table S1: Molecular docking between RSV and SIRT1. Table S2: Molecular docking between RSV and NRF2. Figure S1: The effect of RSV on the survival of PC12 cell at different concentrations. Figure S2: The toxic effect of FAC at different concentrations. Figure S3: The toxic effect of 6‐OHDA at different concentrations. Figure S4: The effect of Fer‐1 on the survival of PD model at different concentrations. Figure S5: Fluorescence quantitative analysis the images from Fig.2H. Figure S6: The effect of RSV on 4‐HNE level in PD cell model. Figure S7: GO enrichment analysis. Figure S8: SIRT1 and NRF2 inhibitors blocked the decrease in 4‐HNE levels induced by RSV in PD mice model. Figure S9: The effect of RSV on SIRT1‐NRF2 signal pathway. [file CNS-31-e70648-s001.docx]

**Supporting Information**

**Groundbreaking Insights into SIRT1/NRF2-Mediated Ferroptosis Inhibition by Resveratrol in Parkinson’s Disease Models**

Qian Zheng,^a,b#^ Dan Huang,^a#^ Liping Zhao,^a^ Xincheng Long,^a^ Qiuxia Tu,^a^ Lingli Song,^a^ Jiaojiao Wang,^a^ Wen Zheng,^a^ Xiaojun Wen,*^a^ Chunlin Zhang,*^a^ and Li Lei*^a^

^a^ Engineering Research Center for Molecular Medicine, School of Basic Medical Science, Guizhou Medical University, Guiyang 550025, China

^b^ Department of Neurology, Affiliated Hospital of Guizhou Medical University, Guiyang, 550001, China

^#^Qian Zheng, and Dan Huang contributed equally to this study.

*Correspondence:

Xiaojun Wen 2547285543@qq.com

Chunlin Zhang [362326474@qq.com](mailto:362326474@qq.com)

Li Lei [1002087851@q](mailto:wen@fjirsm.ac.cn)q.com

**Results**

**Table S1.** Molecular docking between RSV and SIRT1.

**Table S2.** Molecular docking between RSV and NRF2.

**Figure S1.** The effect of RSV on the survival of PC12 cell at different concentrations.

**Figure S2.** The toxic effect of FAC at different concentrations.

**Figure S3.** The toxic effect of 6-OHDA at different concentrations.

**Figure S4.** The effect of Fer-1 on the survival of PD model at different concentrations.

**Figure S5.** Fluorescence quantitative analysis the images from Fig.2H

**Figure S6.** The effect of RSV on 4-HNE level in PD cell model.

**Figure S7**. GO enrichment analysis.

**Figure S8.** SIRT1 and NRF2 inhibitors blocked the decrease in 4-HNE levels induced by RSV in PD mice model.

**Figure S9.** The effect of RSV on SIRT1-NRF2 signal pathway.

**Materials and methods**

***Chemicals and Reagents***

6-OHDA was obtained from Sigma (United States). Prussian Blue Iron Stain kit and MTT Kit were purchased from Solarbio (Beijing, China). JC-1 assay kit, 1-methyl-4-phenyl-1,2,3,6-tetrahydropyridine (MPTP), Liper peroxidation and ROS assay kit were purchased from Beyotime (Shanghai, China). Glutathione (GSH), and malondialdehyde (MDA) were purchased from Nanjing Jiancheng Bioengineering Institute (Nanjing, China). FerroOrange fluorescent probe was purchased from Dojindo Molecular Technology (Japan). EX527 and ML385 were purchased from Aladdin (United States). 4-HNE Enzyme-Linked Immunosorbent Assay Kit was purchased from Fankew (Shanghai, China).

The FTH1, GPX4, NRF2 rabbit monoclonal antibody (HuaBio), SIRT1, TH rabbit monoclonal antibody (Abcam), and GAPDH rabbit monoclonal antibody (Sigma) antibodies were used for immunoblotting. TH rabbit monoclonal antibody (Abcam) was used for immunofluorescence. The primary antibodies were used at 1:1,000 dilution for immunoblotting and 1:100 dilution for Immunofluorescence. The secondary antibodies for immunoblotting analyses were diluted at 1:10,000 (LI-COR, USA), and for Immunofluorescence staining was diluted at 1:200 (Yeasen Biotechnology, Shanghai).

***Molecular docking***

Molecular docking was employed to assess the interactions between RSV and its target proteins identified in the network pharmacology analysis. The 3D structures of the proteins, particularly SIRT1, were predicted using AlphaFold. All compounds were protonated at pH = 7.4, and their 3D structures were generated using Open Babel. AutoDockTools was used to prepare and parametrize both the receptor proteins and ligands. The docking grid files were created with AutoGrid from the Sitemap, and AutoDock Vina (version 1.2.0) was utilized for docking simulations. The optimal docking pose was selected for analysis, and the protein-ligand interaction figure was visualized using PyMOL.

***Cell culture and drug treatment***

Rat pheochromocytoma PC12 cells were purchased from the Cell Bank of Chinese Academy of Sciences (Shanghai, China). The PC12 cells were cultured in Dulbecco’s modified Eagle medium (DMEM) containing 10% fetal bovine serum (FBS) and 50 U/mL each of penicillin/streptomycin. The PC12 cells were inoculated into the plates, pretreated with or without EX527 (10 μM)/ML385 (2 μM) for 1 h, and then treated with RSV (15 μM) for another 2 h. Then, 6-OHDA (200 μM) was added for another day.

***Immunofluorescence of cell***

The cells were washed three times with PBS and fixed with 4% PFA for 15 min at room temperature. After three washes with PBS the cells were permeabilized using 1× PBS with 1% Triton X-100 for 10 min and blocked using 1% BSA for 1 h. The cells were then incubated overnight at 4°C with anti-SIRT1 or NRF2 (1:100) and subsequently with secondary antibodies at room temperature for 2 hours. Fluorescence microscopy with laser scanning confocal imaging was used to capture the results.

***C. elegans culture***

The *C. elegans* strains used in this study included wild-type Bristol N2 (N2), transgenic PD4251, and transgenic BZ555 strains, all obtained from the Caenorhabditis Genetics Center (University of Minnesota). These strains were maintained on nematode growth medium (NGM) plates seeded with Escherichia coli OP50 as the food source at 20 °C, following standard protocols. Fertilized eggs (embryos) were collected from gravid adults using hypochlorite treatment (2% sodium hypochlorite and 0.5 M NaOH). The eggs were incubated for 20 hours at 20 °C in M9 buffer to synchronize the L1 larvae. The synchronized L1 larvae were then transferred to OP50/NGM plates and incubated for an additional 24 hours at 20°C to obtain L3 larvae.

***Evaluation of mouse behavioral changes***

*Open-field (OF) test*

The open-field test was conducted in a square arena (36 cm × 36 cm × 40 cm). Mice were placed in the arena individually and allowed to explore freely for 10 minutes. The movement trajectories of each mouse were recorded by a fixed overhead camera, and the data were analyzed using a video tracking system.

*Pole-climbing test*

The pole-climbing test was performed on a vertical rough-surfaced pole (50 cm height, 1 cm diameter). Mice were placed on top of the pole, and the time taken for each mouse to descend smoothly to the ground was recorded.

*Forced swimming test (FST)*

FST was performed in a transparent glass cylinder (15 cm diameter) filled with 20 cm of water at 23–25°C. Mice were placed in the water for 2 minutes, and the duration of immobility during the last minute of the trial was recorded. The test was repeated three times for each mouse.

***Immunofluorescence of brain slices***

For tissue processing, mice were deeply anesthetized with 1% sodium pentobarbital (0.1 mg/g, i.p.) and perfused with PBS, followed by 4% PFA. The brains were then swiftly excised, fixed in 4% PFA at 4 °C for 24-48 hours, and subjected to dehydration in a 30% sucrose solution for at least 48 hours. The tissue was sectioned into 5 μm slices, washed three times with PBS for 5 minutes, and blocked with 5% bovine serum albumin (BSA) and 0.2% Triton X-100 at room temperature for 2 hours. Sections were incubated overnight at 4 °C with anti-TH (1:100) and subsequently with secondary antibodies at room temperature for 2 hours. Fluorescence microscopy with laser scanning confocal imaging was used to capture the results.

***Western blot (WB) analysis***

Brain tissues from each group were homogenized in RIPA buffer containing phosphatase and protease inhibitors. Protein concentrations were determined using a BCA protein assay kit, and the samples were mixed with loading buffer in a 4:1 ratio before boiling for 3-5 minutes. Equal amounts of protein from each sample were separated by SDS-PAGE and transferred to polyvinylidene difluoride membranes. The membranes were blocked with 5% BSA at room temperature for 1 hour, incubated overnight at 4 °C with primary antibody (1:10,000), and then incubated with secondary HRP-conjugated anti-rabbit antibody (1:10,000) at room temperature for 2 hours. After washing with TBS, protein signals were detected using an infrared imaging system and quantified via densitometric analysis with QuantityOne software (Bio-Rad, Hercules, USA).

***Assessment of*** ***4-HNE levels***

4-HNE level was measured using 4-HNE Enzyme-Linked Immunosorbent Assay Kit. After treatment, brain tissues from mice were homogenized and sonicated in lysis buffer, followed by centrifugation at 12,000 rpm at 4 °C for 30 minutes. The detection was performed according to the kit instructions. The absorbance (OD value) was measured at a wavelength of 450 nm using a microplate reader, and the concentration of mouse 4-HNE in the samples was calculated via a standard curve.

**Table S1.** Molecular docking between RSV and SIRT1.

| **Receptor: SIRT1 (alphafold)** | **Ligand: Resveratrol**  **CAS 501-36-0** | **-6.3kcal/mol** |
| --- | --- | --- |
| **Hydrogen bond interaction** | ILE403 | 2.9Å |
|  | GLU408 | 2.8Å |
|  | ASN409 | 2.9Å |
| **Electrostatic interaction** | \ |  |
|  |  |  |
| **hydrophobic interaction** | \ |  |
|  |  |  |

**Table S2.** Molecular docking between RSV and NRF2.

| **Receptor:** **NRF2 (alphafold)** | **Ligand: Resveratrol**  **CAS 501-36-0** | **-7.1kcal/mol** |
| --- | --- | --- |
| **Hydrogen bond interaction** | GLU518 | 3.2Å |
|  | ASP526 | 3.0Å |
| **Electrostatic interaction** | \ |  |
|  |  |  |
| **hydrophobic interaction** | \ |  |
|  |  |  |


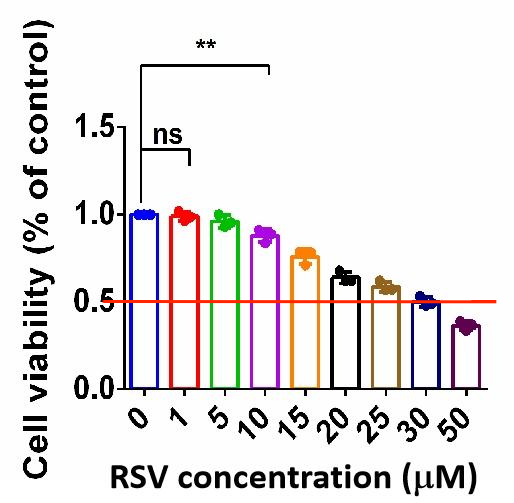


**Figure S1.** The effect of RSV on the survival of PC12 cell at different concentrations (1, 5, 10, 15, 20, 25, 30 and 50 μM). Data expressed as mean ± SD (n = 3 per group). ***P*<0.01, ns: no significant.


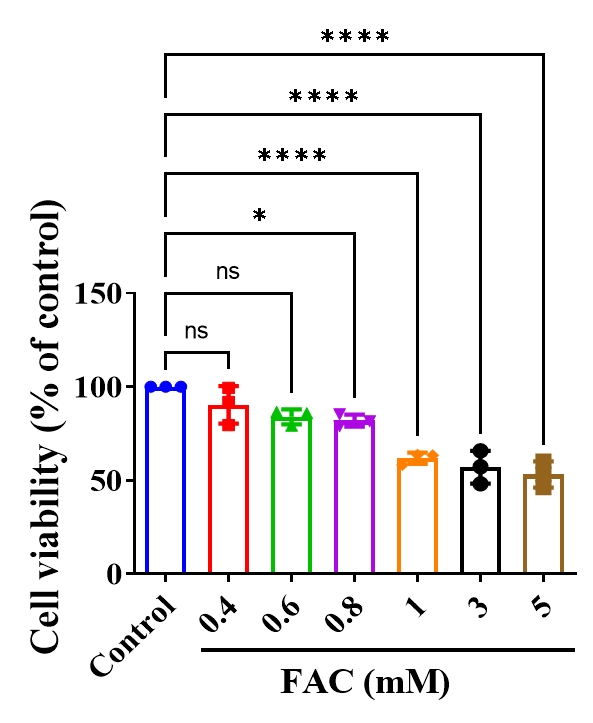


**Figure S2.** The toxic effect of FAC at different concentrations (0.4, 0.6, 0.8, 1, 3 and 5 mM). Data expressed as mean ± SD (n = 3 per group). **P*<0.05, *****P*<0.0001, ns: no significant.





**Figure S3.** The toxic effect of 6-OHDA at different concentrations (50, 100, 150, 200, 250, 300 and 400 μM). Data expressed as mean ± SD (n = 3 per group). ^####^*P*<0.0001.


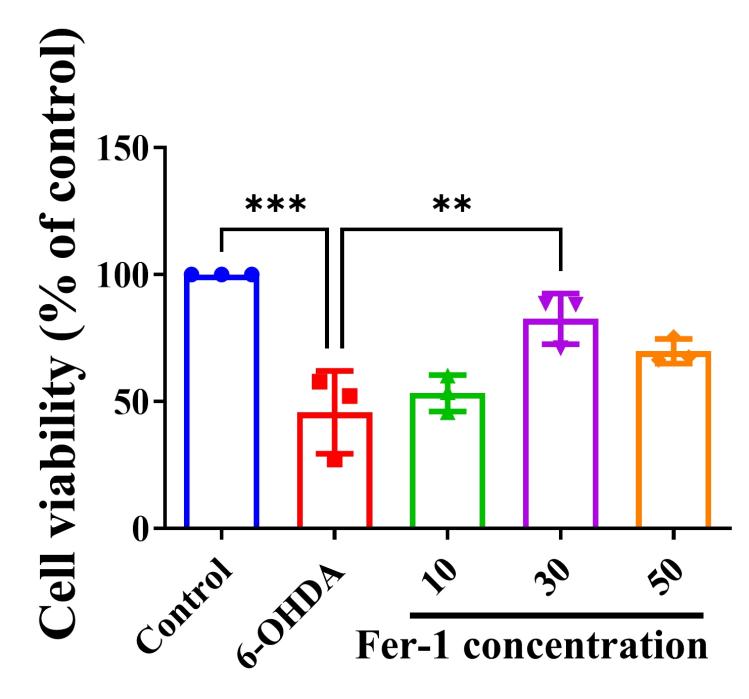


**Figure S4.** The effect of Fer-1 on the survival of PD model at different concentrations (10, 30, and 50 μM).

**
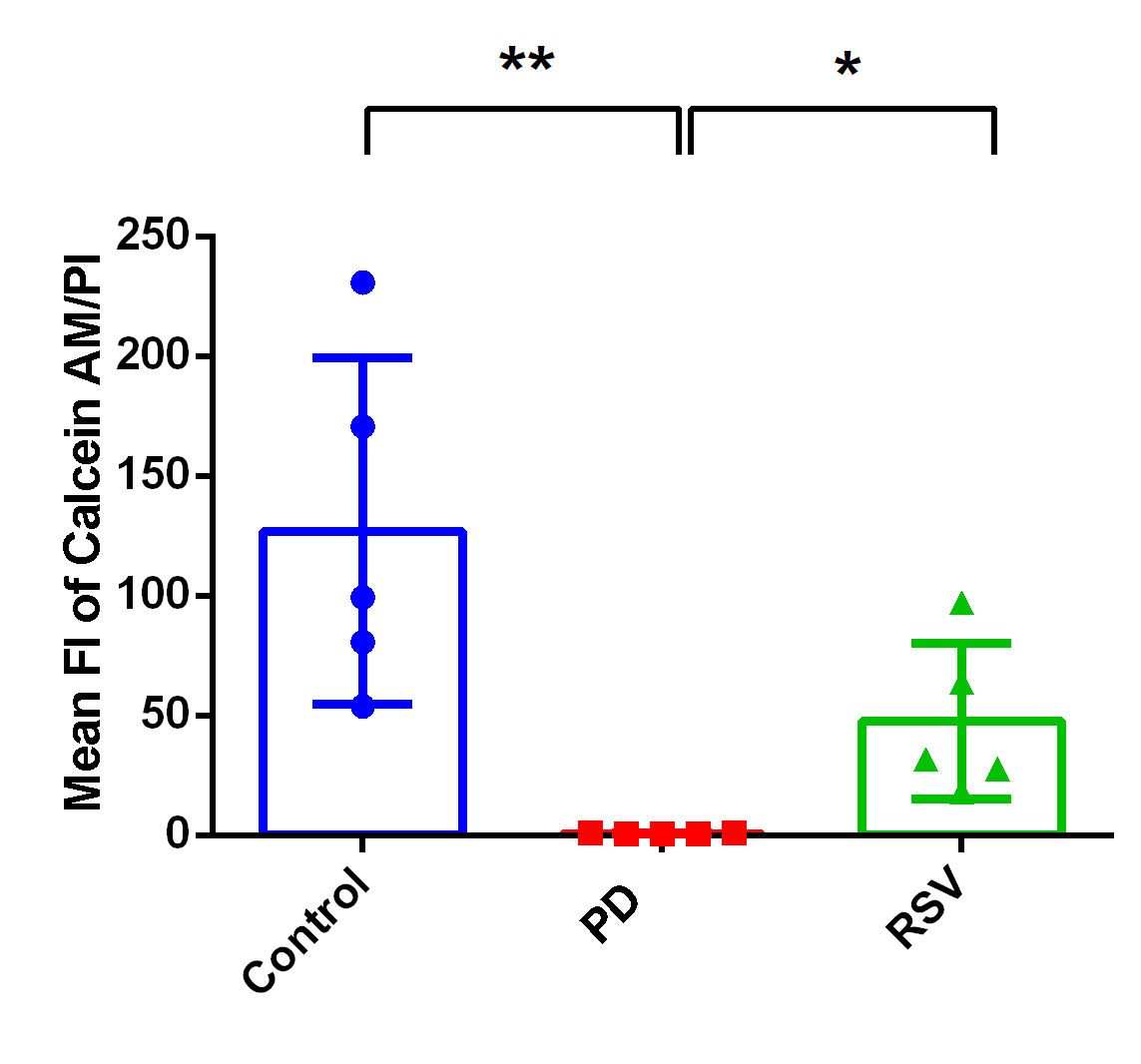
**

**Figure S5.** Fluorescence quantitative analysis the images from Fig.2H (n = 3).


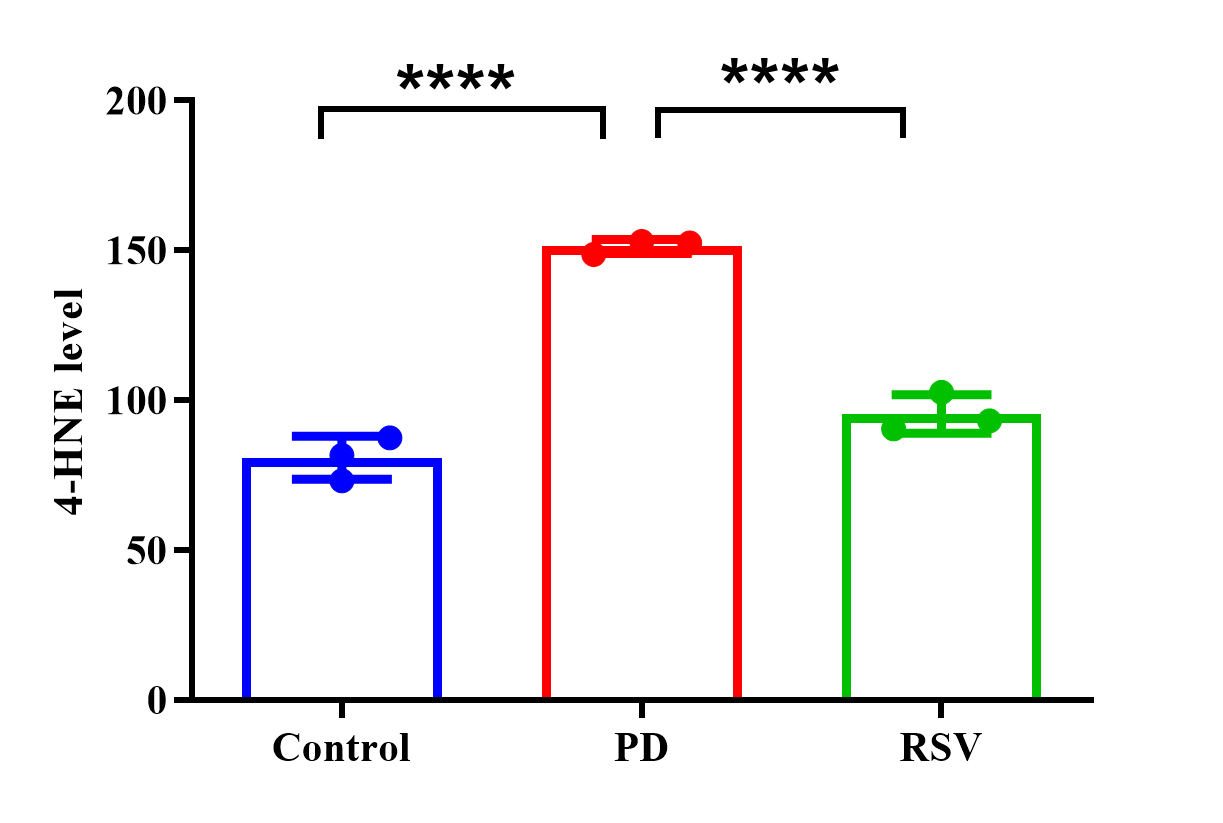


**Figure S6.** The effect of RSV on 4-HNE level in PD cell model**.**


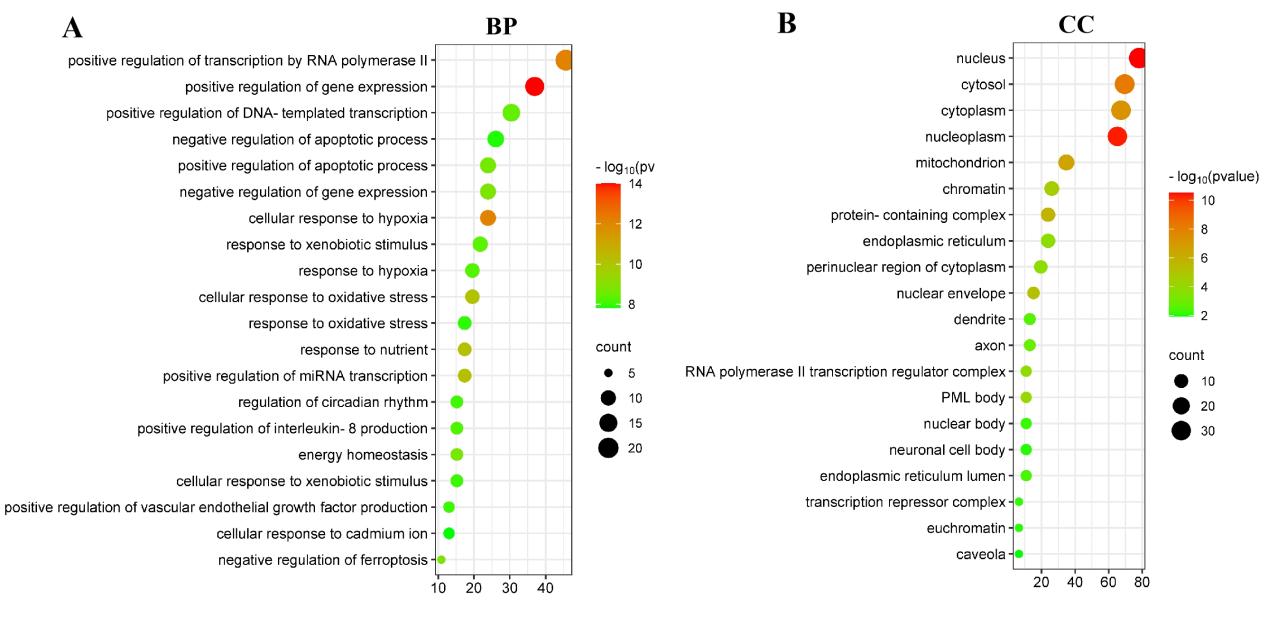


**Figure S7.** (**A-B**) GO enrichment analysis including Biological process (BP), and Cellular component (CC).


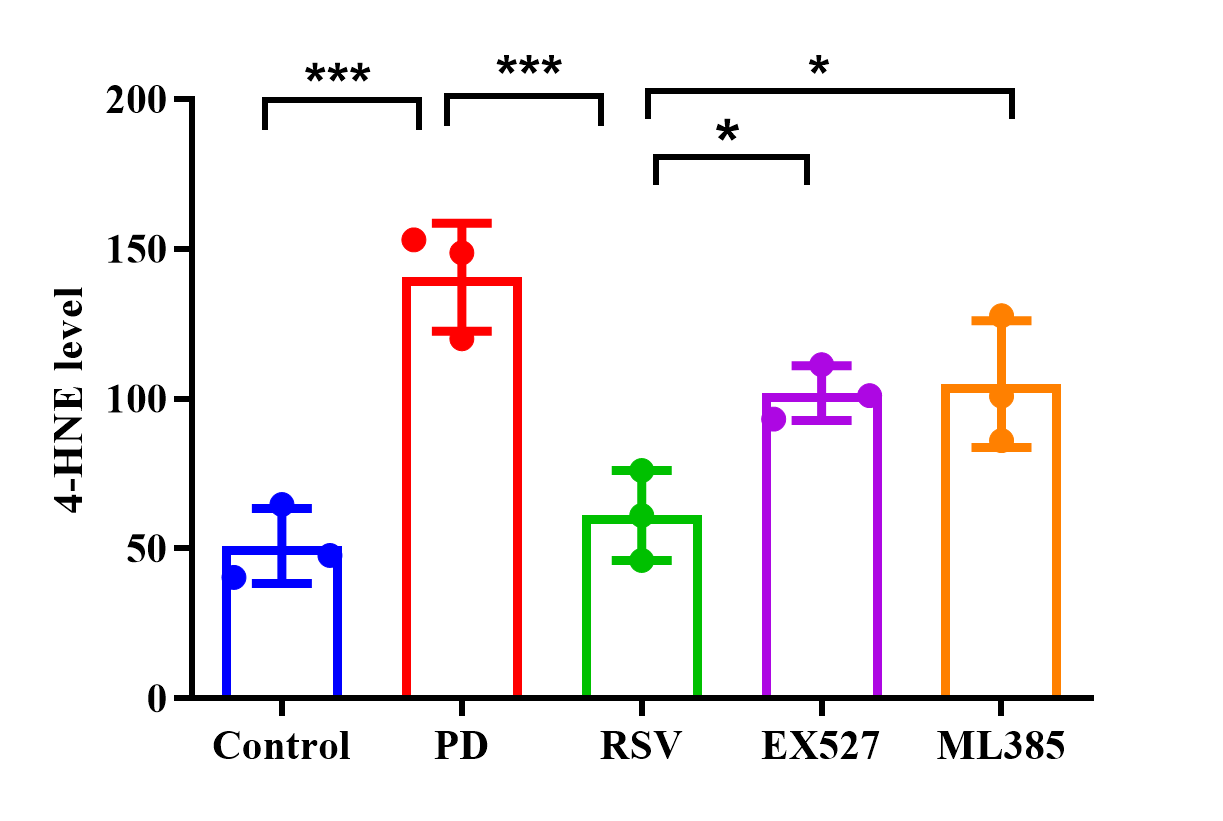


**Figure S8.** SIRT1 and NRF2 inhibitors blocked the decrease in 4-HNE levels induced by RSV in PD mice.


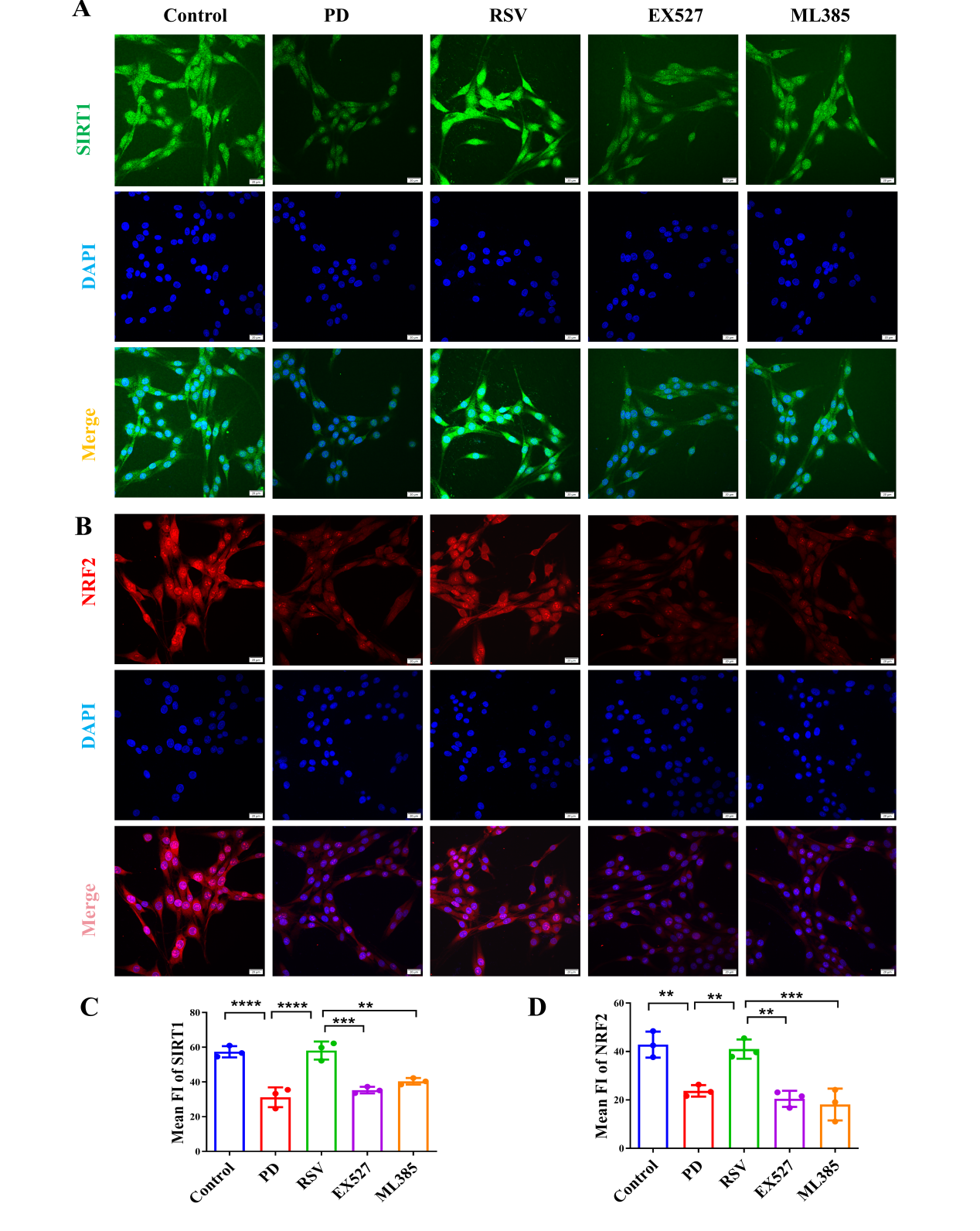


**Figure S9.** The effect of RSV on SIRT1-NRF2 signal pathway. (A, C) The effect of RSV on SIRT1, (B, D) The effect of RSV on NRF2.
